# Expansion of memory Vδ2 T cells following SARS-CoV-2 vaccination revealed by temporal single-cell transcriptomics

**DOI:** 10.1038/s41541-024-00853-9

**Published:** 2024-03-20

**Authors:** Sara Terzoli, Paolo Marzano, Valentina Cazzetta, Rocco Piazza, Inga Sandrock, Sarina Ravens, Likai Tan, Immo Prinz, Simone Balin, Michela Calvi, Anna Carletti, Assunta Cancellara, Nicolò Coianiz, Sara Franzese, Alessandro Frigo, Antonio Voza, Francesca Calcaterra, Clara Di Vito, Silvia Della Bella, Joanna Mikulak, Domenico Mavilio

**Affiliations:** 1 Laboratory of Clinical and Experimental Immunology, IRCCS Humanitas Research Hospital, Milan, Rozzano Italy; 2 Department of Biomedical Sciences, Humanitas University, Milan, Pieve Emanuele Italy; 3 Department of Medical Biotechnology and Translational Medicine, University of Milan, Milan, Italy; 4 Department of Medicine and Surgery, University of Milan-Bicocca, Monza, Italy; 5 Institute of Immunology, Hannover Medical School (MHH), Hannover, Germany; 6 Institute of Systems Immunology, Hamburg Center for Translational Immunology (HCTI), University Medical Center Hamburg-Eppendorf, Hamburg, Germany; 7 Department of Biomedical Unit, IRCCS Humanitas Research Hospital, Milan, Rozzano Italy

**Keywords:** RNA vaccines, Innate immunity

## Abstract

γδ T cells provide rapid cellular immunity against pathogens. Here, we conducted matched single-cell RNA-sequencing and γδ-TCR-sequencing to delineate the molecular changes in γδ T cells during a longitudinal study following mRNA SARS-CoV-2 vaccination. While the first dose of vaccine primes Vδ2 T cells, it is the second administration that significantly boosts their immune response. Specifically, the second vaccination uncovers memory features of Vδ2 T cells, shaped by the induction of AP-1 family transcription factors and characterized by a convergent central memory signature, clonal expansion, and an enhanced effector potential. This temporally distinct effector response of Vδ2 T cells was also confirmed in vitro upon stimulation with SARS-CoV-2 spike-peptides. Indeed, the second challenge triggers a significantly higher production of IFNγ by Vδ2 T cells. Collectively, our findings suggest that mRNA SARS-CoV-2 vaccination might benefit from the establishment of long-lasting central memory Vδ2 T cells to confer protection against SARS-CoV-2 infection.

## Introduction

The vaccination campaign against Severe Acute Respiratory Syndrome Coronavirus 2 (SARS-CoV-2), which causes Coronavirus Disease 2019 (COVID-19), has expanded worldwide. The Pfizer-BioNtech *BNT162b2* was the first vaccine to receive the US Food and Drug Administration (FDA) approval in December 2020. *BNT162b2* is a nucleoside-modified RNA vaccine that encodes a membrane-anchored SARS-CoV-2 full-length Spike-protein. The *BNT162b2* vaccine shows high efficiency in preventing symptomatic COVID-19^1–4^. However, over time, the vaccine’s effectiveness decreases, and repeated doses of administration have been recommended^5–7^.

Factors contributing to protection after vaccinations are not fully known. Humoral immune responses have been identified to provide crucial protection against infection, with the level of plasma neutralizing antibodies (Abs) being a key predictor of efficacy against SARS-CoV-2 infection^8^. Additionally, T cells exhibit robust recall responses following booster vaccination, supporting the functional nature of mRNA vaccine-induced T cell memory^9–13^. Notably, SARS-CoV-2 infection may elicit a “cellular sensitization without seroconversion” since some individuals develop efficient T cellular responses without detectable virus-specific Abs^9,14,15^. In this scenario, a comprehensive characterization of the T cell response following vaccination, including unconventional T cells, and their role in post-vaccine protection, is less explored.

Gamma delta (γδ) T cells are unconventional cytotoxic T lymphocytes, comprising approximately 1–5% of peripheral blood (PB) CD3^+^ T cells in homeostatic conditions^16^. Human γδ T cells are primarily categorized into two types based on the Vδ-TCR region: Vδ1 and Vδ2 T cells. While Vδ2 T cells make up the largest population of γδ T cells in the PB, Vδ1 T cells usually reside in peripheral tissues including the lung, liver, and gut^16–18^. γδ T cells provide protection against tumors, as well as bacterial and viral pathogens, including Human Cytomegalovirus (HCMV) and Human Immunodeficiency Virus (HIV), through the rapid release of cytokines (IFNγ, TNF) and direct killing of infected cells^16,18–23^.

Distinctive expression patterns of CD27 and CD45RA/RO surface markers, identify different γδ T cell subsets, primarily classified as naïve (T_N_; CD27^+^CD45RA^+^), central memory (T_CM_; CD27^+^CD45RA^−^), effector memory (T_EM_; CD27^−^CD45RA^−^), and highly differentiated effector memory (T_EMRA_; CD27^-^CD45RA^+^). These subsets differ in proliferative capacities, effector functions, and resistance to cell death in response to antigens and/or cytokine stimulation^16,18,24–26^. Nevertheless, it remains unclear whether γδ T cells exhibiting memory phenotype are inherently reprogrammed cells from prior activation or antigen exposure, leading to a more effective secondary immune response.

The early years of the SARS-CoV-2 pandemic have highlighted several reports linking COVID-19 to the activation of γδ T cells, their differential transition, and their significant inhibitory potential on SARS-CoV-2 replication^27–29^. Additionally, recent flow cytometry analyses have demonstrated the activation of various γδ T cell subsets in response to COVID-19 vaccination during pregnancy^30^. However, the role of γδ T cells in the immunological response to COVID-19 vaccination requires further investigation.

Here, we generated single-cell RNA sequencing (scRNA-seq) data paired with single-cell γδ-TCR data to track γδ T cells following repeated vaccinations with the Pfizer-BioNTech *BNT162b2*. Our analysis encompassed various aspects of the γδ T cell response, including response magnitude, altered gene expression profiles, biological pathway activation, and the composition of the γδ-TCR repertoire. We observed that the second exposure of γδ T cells to the vaccine revealed, particularly in Vδ2 T cells, a gradual enhancement of their effector potential, associated with clonal expansion and the formation of memory states. In vitro experiments confirmed that the second challenge of Vδ2 T cells with SARS-CoV-2 spike-peptides led to a more robust effector response.

Overall, our findings emphasize that the early response of Vδ2 T cells to mRNA SARS-CoV-2 vaccine contributes to immunization. Furthermore, our study introduces the concept of Vδ2 T cell memory generation characterized by an enhanced effector potential following the initial SARS-CoV-2 vaccination.

## Results

### Single-cell mapping of γδ T cell activation following SARS-CoV-2 vaccination

**Fig. 1:**
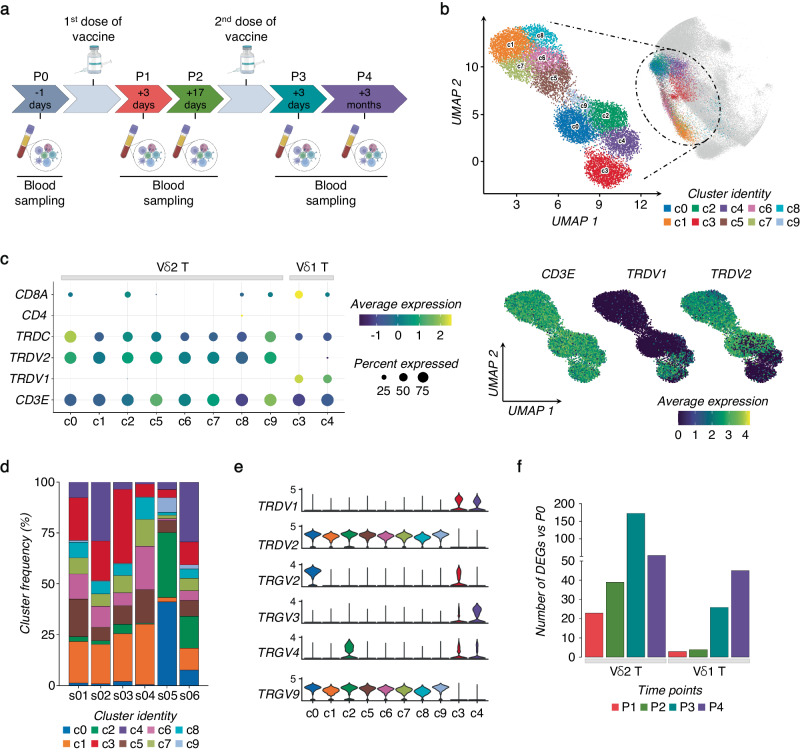
Activation of γδ T cells following SARS-CoV-2 vaccination. **a** Schematic overview of the experimental design. **b** *UMAP* clustering projection of the integrated PB γδ T cells from all subjects (s01-s06; a total of 12640 γδ T cells). **c** The dot plot (left panel) and feature plots (right panel) show the expression of canonical markers used for the annotation of γδ T cell subtypes. **d** The bar plot shows the frequency (%) of cell cluster (c0-c9) distribution across all subjects. Cell numbers were normalized to the total number of cells per subject loaded independently from the time point. **e** The violin plots show the expression of the main TCR γ chains (*TRGV*) associated with either δ1 (*TRDV1*) or δ2 (*TRDV2*) chains per cluster. **f** The bar plot shows the number of identified DEGs across different time points (P1-P4) after vaccination, compared to pre-vaccine P0. For all figures, DEGs were defined as follows: (i) absolute value of average |Log_2_- FC| ≥ 0.25 for up- and down-regulated genes; (ii) adjusted *P* values (adj.p) ≤ 0.05, and (iii) detected not less than 10% of cells (min.pct ≥ 10%).

To investigate the effect of SARS-CoV-2 vaccine exposure on γδ T cells, we performed a longitudinal scRNA-seq study on 6 SARS-CoV-2–naïve volunteers vaccinated with two doses of the Pfizer-BioNTech *BNT162b2* mRNA vaccine (Fig. 1a and Supplementary Fig. 1a). PB samples were collected 1 day before (P0), 3- and 17-days after prime vaccination (P1 and P2, respectively), and 3-days and 3-months following vaccine-boost (P3 and P4, respectively). γδ T cells were identified by their expression of CD3 (*CD3E*) and TCR canonical δ constant (*TRDC*) and variable δ (*TRDV*) region-encoding segment (Supplementary Fig. 1b). Initially identified clusters of γδ T cells were poorly-resolved, necessitating further re-clustering to obtain purified and separable groups of γδ T cells (Method section). In total, we refined 12640 γδ T cells that Uniform Manifold Approximation and Projection (*UMAP*) analysis resolved into 10 different γδ Τ cell clusters (c0-c9) as visualized by the top 10 differentially expressed genes (DEGs) (Fig. 1b, c, and Supplementary Fig. 1c). Specifically, we identified clusters 3 and 4 (c3, c4) as Vδ1 T cells expressing *TRDV1* segments; instead, clusters c0-c2 and c5-c9, were identified as Vδ2 T cells as those expressing *TRDV2*. The relative frequencies of each cluster in the 6 samples are represented in Fig. 1d. As expected^31^, Vγ9 emerged as the main Vγ chain (*TRGV*) associated with Vδ2-enriched clusters (Fig. 1e). On the other hand, previously defined as adaptive-like Vγ9^neg^Vδ2 T cells^32^, were enriched in c0 and c2. In fact, cells in c0 and c2 present high expressions of Vγ2 and Vγ4, respectively. These cells are rarely present in adults PB^32^, and were mainly presented in one of the analyzed individuals. Moreover, two main Vγ3 and Vγ2 chains were linked to Vδ1-enriched T cell clusters.

**Fig. 2:**
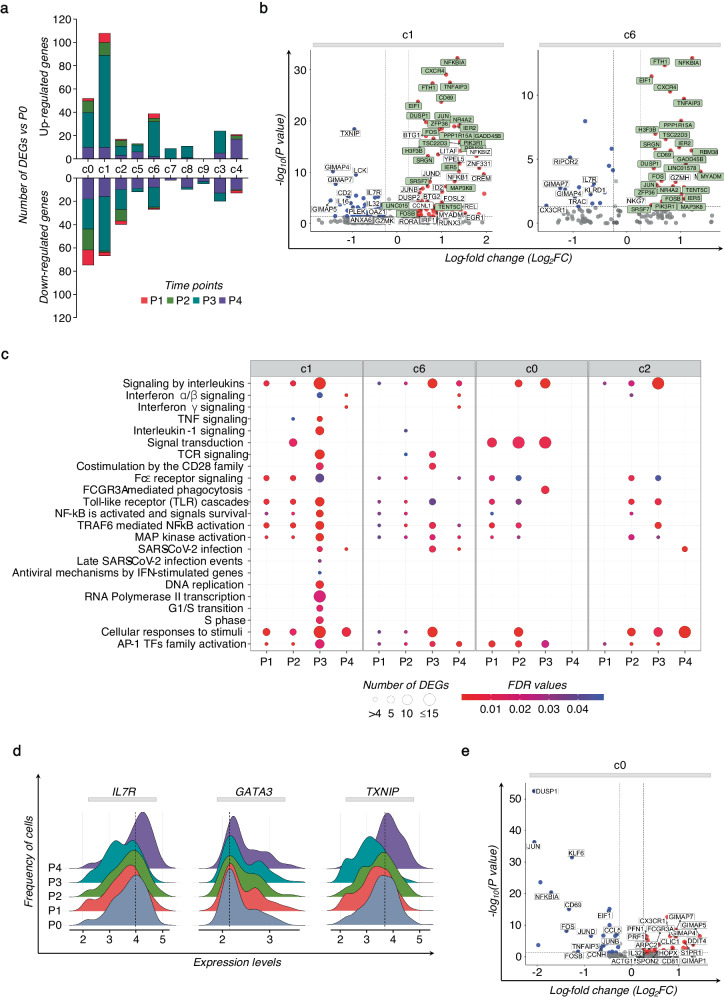
Identity of the specific γδ T cell cluster activation following SARS-CoV-2 vaccination. **a** The bar plot shows the number of up- and down- modulated DEGs at different post-vaccine time points (P1-P4) calculated for each γδ T cell cluster, compared to the pre-vaccine P0. **b** The volcano plots highlight DEGs of the specific Vδ2 T cell clusters c1 and c6, resulting in the highest number of DEGs at P3. Green boxes indicate common genes expressed in clusters c1 and c6. Red dots indicate genes up-regulated in P3 *vs* P0, and blue dots indicate up-regulated genes in P0 *vs* P3. **c** The dot plot shows a selection of significantly enriched pathways with FDR values <0.05, identified among DEGs at each time point (P1-P4) for specific Vδ2 T cell clusters (c0-c2, c6) using the *Reactome* pathway browser. Dots are colored by FDR values and sized by the number of DEGs enriched in each pathway. **d** The ridge plots show the expression levels (x-axis, log-UMI) and the frequency of cells (y-axis) of three DEGs found in cluster c1 at P4 compared to pre-vaccine P0. Null gene expression cells were excluded from the analysis. The dotted line indicates the gene expression level corresponding to the highest cells’ frequency at the baseline (P0). **e** The volcano plots highlight DEGs of the Vδ2 T cell cluster c0 at P3 compared to P0. Red dots indicate genes up-regulated in P3 *vs* P0, and blue indicate genes up-regulated in P0 *vs* P3.

The relative abundance of Vδ1 and Vδ2 T cell clusters in each individual, and at different time points did not display substantial changes (Supplementary Fig. 1d). However, significant variations were observed in their transcriptome profiles, in terms of kinetics and amplitude of DEGs, before and after immunization. Specifically, minor changes were detected in the transcriptome of Vδ1 and Vδ2 T cells early after the prime-vaccine at P1 and P2. Instead, their transcriptional profile substantially changed after the vaccine-boost, suggesting that the first vaccine dose primed γδ T cells into a “trained” status that the boost tuned to a higher responsiveness (Fig. 1f). We then investigated the features and dynamic changes in individual γδ T cell clusters in relation to DEGs and to the underlying transcriptional programs (*Reactome*). Heterogeneous responses were observed across different Vδ2 T cell clusters (Fig. 2a). Clusters c1 and c6 exhibited early induction of *CD69* at P1, and various members of the AP-1 transcription factor (TF), particularly prominent in c1 (Supplementary Fig. 2a). Clusters c1 and c6 were also characterized by a higher response after the vaccine booster, sharing different activated profiles, including AP-1 TFs (i.e, *JUN*, *JUNB, JUND, FOS*, *FOSB*, *FOSL2*) and genes related to the NF-κB activities (i.e., *NFKBIA*, *NFKB1, TNFAIP3, MAP3K8*) (Fig. 2b). In fact, pathway enrichment analysis based on all DEGs calculated compared to P0 resulted in a notable transcriptional reprogramming marked in c1, and involving AP-1 and NF-κB TFs, interleukins (ILs), interferons (IFNs), and TNF pathways, and the TCR-complex signaling engagement (Fig. 2c). Cascades of Toll-like receptors (TLRs) and pathways regulating the response to SARS-CoV-2 infection show progressive activation in c1 and c6. Importantly, the activation pattern in c1 after the second vaccine dose indicated a proliferation signature, reprogramming of metabolism and the memory T cell preservation (*TXNIP, GATA3*, and *IL7R*) (Fig. 2c, d)^33–36^.

Vδ2 T cell activation in c0 and c2 were related to their cytotoxic potential, as evidenced by increased DEGs such as *FCGR3A*, *PRF1*, *ACTG1*, *S100A4*, and *CX3CR1*, along with the down-modulation of AP-1 TFs, distinct from c1 and c6 (Fig. 2c, e). The remaining clusters of Vδ1 and Vδ2 T cells demonstrated reduced biological pathway activation in response to vaccine stimuli (Supplementary Fig. 2b).

Based on the heterogeneous response found among different clusters of γδ T cells, we reasoned that γδ T cells might exhibit varying responsiveness to SARS-CoV-2 vaccine strengthened upon immunization. Moreover, we identify AP-1 TFs as potential drivers that contribute to the increased Vδ2 T cell response at P3 after the second vaccine dose.

### Transcriptional changes in Vδ2 T cells following SARS-CoV-2 vaccination

**Fig. 3:**
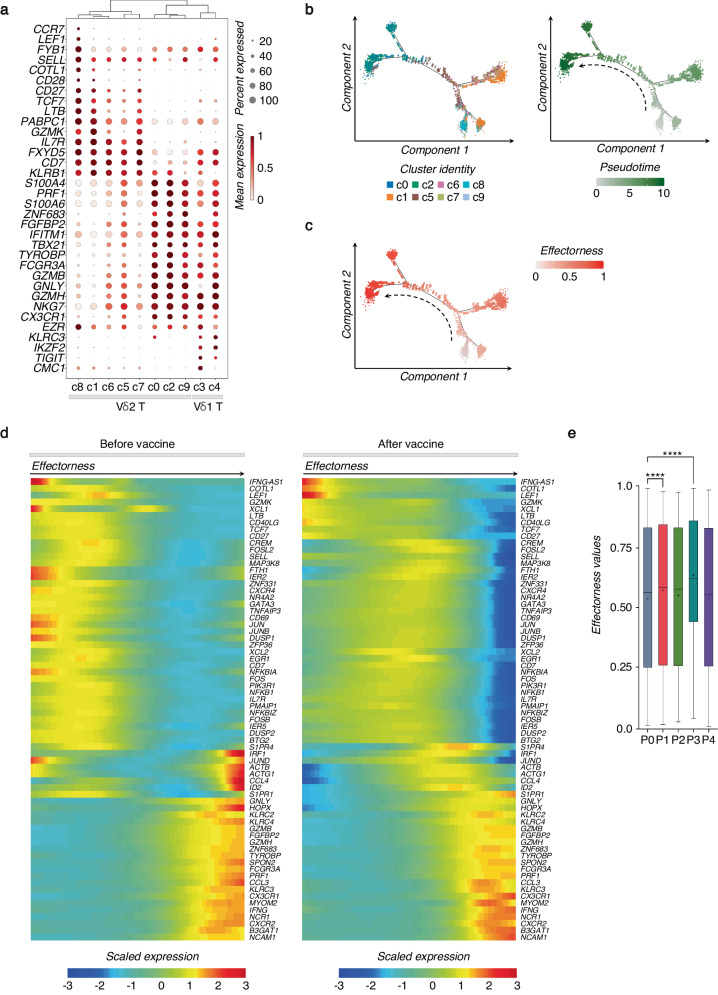
SARS-CoV-2 vaccination shapes the effectorness of Vδ2 T cells. **a** The dot plot shows the expression of selected genes for each γδ T cell cluster at all the time points (P0-P4), together. Dots are colored by the average expression of each gene scaled across all clusters and sized by the percentage of cells within a cluster (min.pct ≥ 10%). Clusters are ordered according to the hierarchical clustering shown by the dendrogram. **b** Pseudotime trajectory of Vδ2 T cells, where each cell is colored by its cluster identity (c0-c2, c5-c9) (left panel) or its pseudotime value (right panel). **c** Effectorness gradient mapped on the pseudotime trajectory of Vδ2 T cells, with each cell colored by its effectorness value. **d** Heatmap of selected genes variable along the pseudotime trajectory (from Monocle) calculated before vaccine at P0 (left panel) or after all (P1 + P2 + P3 + P4) vaccination time points (right panel). The x-axis represents cells ordered by pseudotime (from left to right), and different colors correspond to the scaled (Z-scored) expression of each gene in each cell. **e** The box plot shows effectorness values at different time points (P0-P4), with the median represented by a line and the mean indicated by a “+” across the boxes. The vertical bars show the min-max range distribution. For the statistical analysis, the unpaired nonparametric Dunn’s test was used to perform multiple comparisons *vs* the control P0, statistically significant comparisons are represented as *P* values (*): ****P < 0.0001.

We found that the main source of variation across all clusters can be imputed to the differentiation phenotypes of γδ T cells regardless of the vaccination (Fig. 3a). Indeed, the cells in c8 with T_N_ profile express higher levels of *CCR7*, *LEF1*, *FYB1*, *SELL*, and *COTL1*^37,38^. Cells in c1 show T_N_/T_CM_ profile that share several markers including *IL7R*, *TCF7*, and *CD27*. While cells expressing cytotoxic-related genes (i.e., *GZMA*, *GZMB*, and *PRF1*) represent γδ T cells that exhibit T_EM_/T_EMRA_ patterns. We observed that T_CM_ Vδ2 T cell clusters, enclosed in c5-c7, show a gradual transition to the T_EM_ profile. Compared to more heterogeneous Vδ2 T cells, Vδ1 T cells aggregate in two clusters with T_EM_/T_EMRA_ cytotoxic outlines that mainly differ in Vγ chain usage, expression of *KLRC2* (NKG2C), and TFs expression such as *ZNF683* (Supplementary Fig. 1c), thus indicating their diverse functional commitment.

To further investigate relationships between Vδ2 T cell clusters, we applied pseudotime analysis on Vδ2 T cells including all the time points (Fig. 3b and Supplementary Fig. 3). The trajectory of Vδ2 T cells started from highly enriched T_N_/T_CM_ markers (e.g., *COTL1*, *GZMK*, *LEF1*, *CD27*, and *IL7R*) and finished with the markers of highly differentiated cells expressing *B3GAT1* (CD57), *NCAM1* (CD56), *FCGR3A* (CD16), and *CX3CR1*, cytotoxic molecules (*GZMB*, *GZMH*, *PRF1*, *GNLY*), cytokines and chemokines (*IFNG*, *CCL3-4*, *CCL4L2*). Based on the pseudotime analysis, we observed that Vδ2 T cell clusters do not comprise discrete subpopulations but instead represent interrelated multiple differentiation stages with a continuous advancement from T_N_ and T_CM_ profiles, toward cells with T_EM_ phenotype (Fig. 3c). We reasoned that the observed Vδ2 T cell continuum reflects the potential to initiate a rapid effector response upon stimulation. We term this property “effectorness”, based on what has been observed for CD4^+^ T cells^39^. To assess if the vaccine influences impacts the effectorness of Vδ2 T cells, we generated pre- and post-vaccine pseudotime trajectories (Fig. 3d). To define the effectorness score, we scaled the pseudotime values for each trajectory to the range [0; 1] (Method section). In both trajectories, we found equivalent routes defined by initial genes corresponding to the T_N_ profile [0 = T_N_] and terminal genes defining T_EM_ cells [1 = T_EM_], indicating that the effectorness gradient is detectable before and after vaccination. We observed that the vaccine increases the effectorness values of cells with T_N_/T_CM_ phenotype. Indeed, cells with a T_N_/T_CM_ profile increased their effectorness by shifting to the right within the effector range after vaccination. Notably, AP-1 family genes, including *FOSL2*, *JUN, JUNB*, *FOS*, and *FOSB* are the main TFs associated with the increased effectorness. To support the notion that Vδ2 T cell effectorness increases upon vaccination, we calculated the cell distribution at different time points based on cell effectorness (Fig. 3e). We found an increased number of cells with an enhanced effectorness from P1 to P3, thus suggesting that the effectorness of Vδ2 T cells is a consequent of their vaccination history.

As for Vδ1 T cells, their reduced pathway activation, compared to Vδ2 T cells, hinders a similar effectorness annotation. These findings imply that the distinct TCR repertoire-phenotype states of Vδ1 and Vδ2 T cells before vaccination are associated with their differing responses to the vaccine.

### SARS-CoV-2 vaccinations lead to increased effector response and memory phenotype of Vδ2 T cells

**Fig. 4:**
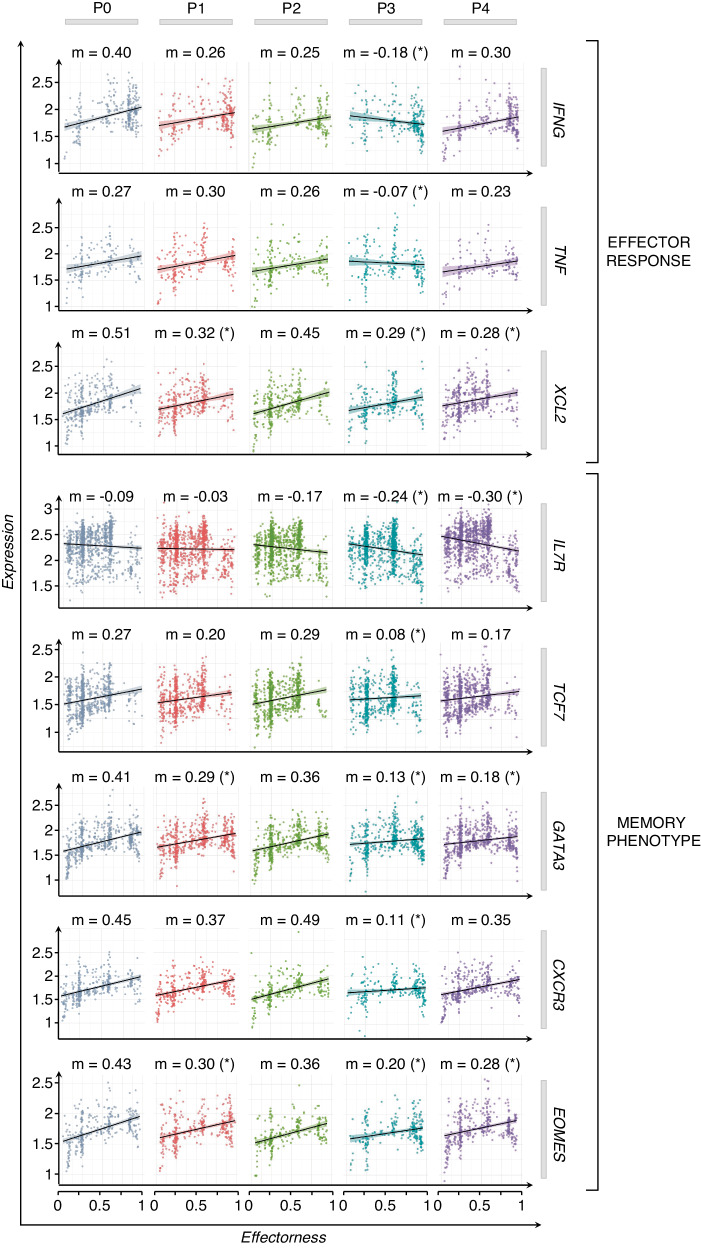
The interaction between effectorness and vaccination regulates Vδ2 T cell response. Plots of gene expression (y-axis, log-normalized UMIs) as a function of effectorness (x-axis), stratified by time points (P0-P4). Examples of significant cross-reactive genes, selected for their activation (top panels), effector (middle panels), and memory-associated phenotype (bottom panels) of Vδ2 T cells. Each dot represents a single cell with a gene expression value >0. The “m” value above each graph represents the coefficients of linear regression. Significance was calculated using a *t*-test, and only significant *P* value*s* (*) are shown (*P* < 0.05).

Our results indicate that the vaccine shapes the effectorness of Vδ2 T cells. To further characterize the role of SARS-CoV-2 vaccination in shaping the effectorness of Vδ2 T cells at different time points, we used a multiple linear regression (MLR) model to predict gene expression with effectorness, vaccination, and their reciprocal interaction as independent variables (Methods section)^39^. With this approach, we found 179 genes whose expressions were significantly modulated by effectorness and vaccination acting jointly due to their cross-reactive effect (Supplementary Table 1). Among them, we found genes with a high impact on the effector response such as *IFNG*, *TNF*, and *XCL2*, and several genes crucial for durable T_CM_ phenotype preservation including *IL7R*, *TCF7*, *GATA3*, *CXCR3*, and *EOMES*^34,35,40–42^. Profiling the expression of these genes over different time points revealed their progressive correlation with the vaccine stimulations (Fig. 4). For example, genes regulating effector response (*IFNG*, *TNF*, and *XCL2*) increased (as indicated by the angular coefficient ‘*m*’) after the first dose of the vaccine; however, their magnitude peaked after the second dose. Similar changes have been observed for the memory-associated genes (*IL7R*, *TCF7*, *GATA3*, *EOMES, CXCR3*) that, moreover, resulted in a more durable memory phenotype detected in Vδ2 T cells over 3 months after vaccination. These data clearly show the critical role of the first vaccination that elicits enhanced immune response and transcriptional changes of Vδ2 T cells after the second dose.

Overall, the transcriptomic status of Vδ2 T cells during SARS-CoV-2 vaccination is influenced by the mutual interaction between effectorness and the number of vaccinations, leading to an enhancement of their effector potential associated with memory profile.

### Adaptation of γδ-TCR repertoire to SARS-CoV-2 vaccination

**Fig. 5:**
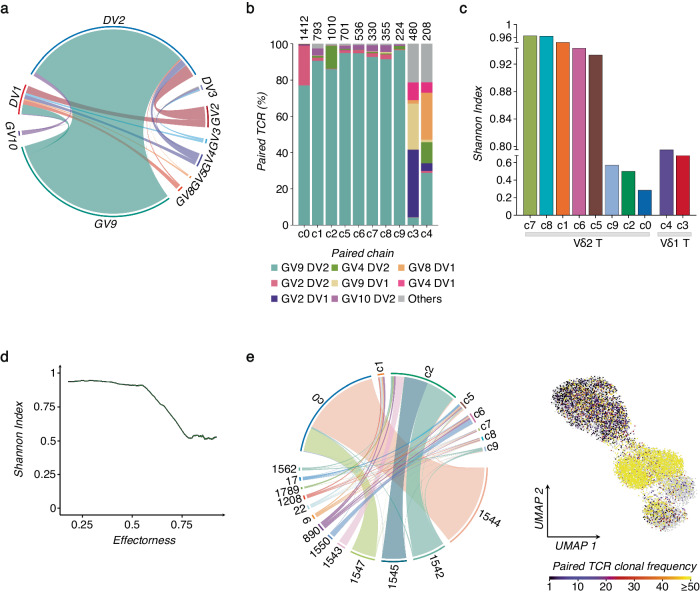
Single cell γδ-TCR repertoire analysis. **a** Chain pairing of different GVs and DVs are displayed as chord diagrams, where ribbons connecting chains are proportional to the number of paired GV/DV chains. **b** The bar plot shows the frequency of the most representative paired GV/DV chains for each cluster (c0-c9). The total numbers of paired GV/DV chains per cluster are indicated at the top of each bar, and the most abundant pairs are color-coded. **c** γδ-TCR repertoire diversities were estimated by the normalized Shannon Index, ranging from 0 (completely monoclonal) to 1 (completely polyclonal). The bar plot shows the Shannon Index of each cluster (c0-c9). **d** Plot of the normalized Shannon Index (y-axis) as a function of the effectorness gradient (x-axis) of Vδ2 T cells, calculated using a sliding window approach (Methods). **e** The chord diagram (left panel) shows the overlap of highly expanded clones (≥50 cells) across different Vδ2 T cell clusters (c0-c2, c5-c9). Spatial visualization on the *UMAP* (right panel) of all identified GV/DV paired clones Vδ2 T cells, scaled from unique clonotypes to highly expanded clones (≥50).

To gain a deeper understanding of the γδ T cell response to the vaccine, we linked our scRNA-seq data with the individual γδ-TCR clones generated as described in the Methods section. Among TRD or TRG sequences, detected in all barcoded cells, we found TRG and TRD pairs in about 40% of all single γδ T cell transcriptomes. For further analysis, we considered only the paired γδ TCRs. The diversity and distribution of paired *DV* and *GV* genes are shown in Fig. 5a, b, and Supplementary Fig. 4a. As expected, *GV9*-*DV2* pairs represented the most abundant combination among all Vδ2 T cell clusters in all donors. On the other hand, *DV1* mainly paired with *GV2*, *GV9*, and *GV8*. We then investigated the diversity of γδ-TCR clonotypes across all cell clusters, defined by *UMAP,* by calculating the Shannon index, which ranges from 1 (polyclonal) to 0 (monoclonal) (Fig. 5c). The two Vδ1 T cell-enriched clusters, c3-4, in concordance with their high effector phenotypes, show more oligoclonal features with a relatively low Shannon index. We observed that the gradual clonal expansion aligned with the progressive differentiation states of Vδ2 T cells, accompanied by increasing effectorness (Fig. 5c, d). Moreover, clonal Vδ2 T cell expansion correlates with the persistence of discrete subpopulations with heterogeneous phenotypes. Indeed, approximately 15% of the clonal composition of the most expanded (≥50 cells) *DV2* clones overlaps with the clusters displaying T_CM_ profile (Fig. 5e and Supplementary Fig. 4b).

Overall, these data indicate that Vδ2 T cell clonal expansion is accompanied by increased effectorness and the persistence of T_CM_ phenotype.

**Fig. 6:**
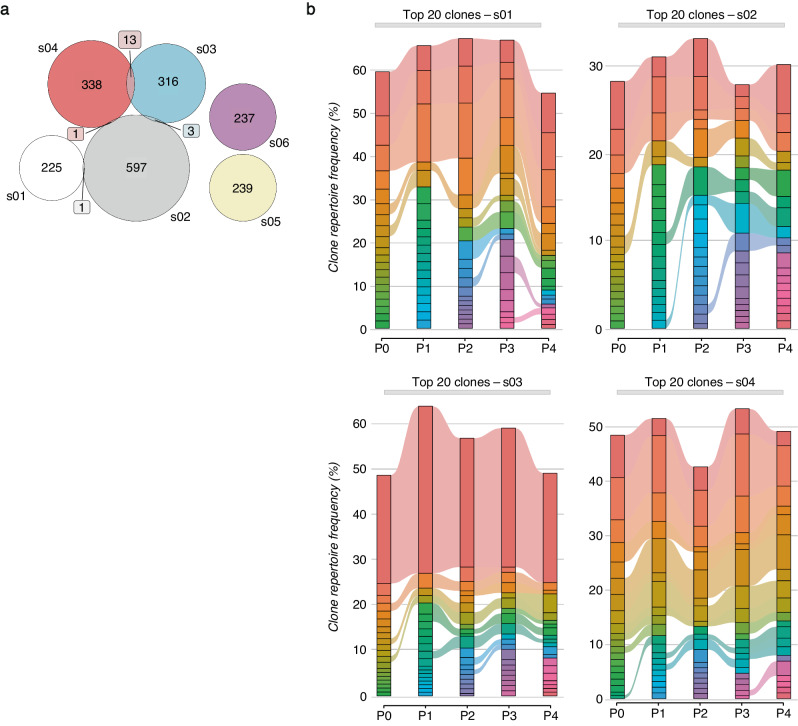
SARS-CoV-2 vaccine shapes γδ-TCR repertoire. **a** The Venn diagram shows the counts (colored box) of overlapped paired γδ-TCR clonotypes among different subjects (s01-s06); the numbers represent subject-unique or shared γδ-TCR clonotypes among different individuals. **b** Longitudinal tracking of the 20 top-most expanded γδ-TCR clones for selected subjects (s01-s04), stratified for different time points (P0-P4). Each stratum represents a unique γδ-TCR clonotype highlighted by a different color. The colored bands between columns represent shared clones among time points.

Building upon this evidence, we tracked the fate of individual γδ-TCR clonotypes concerning the vaccination timeline. Among the total analyzed clonotypes, about 1% overlapped between more than one subject (Fig. 6a and Supplementary Fig. 5a). On the other hand, longitudinal tracking of the top 20 abundant clones in all analyzed time points revealed that the γδ-TCR repertoires in most subjects differed from those before vaccination (Fig. 6b).

**Fig. 7:**
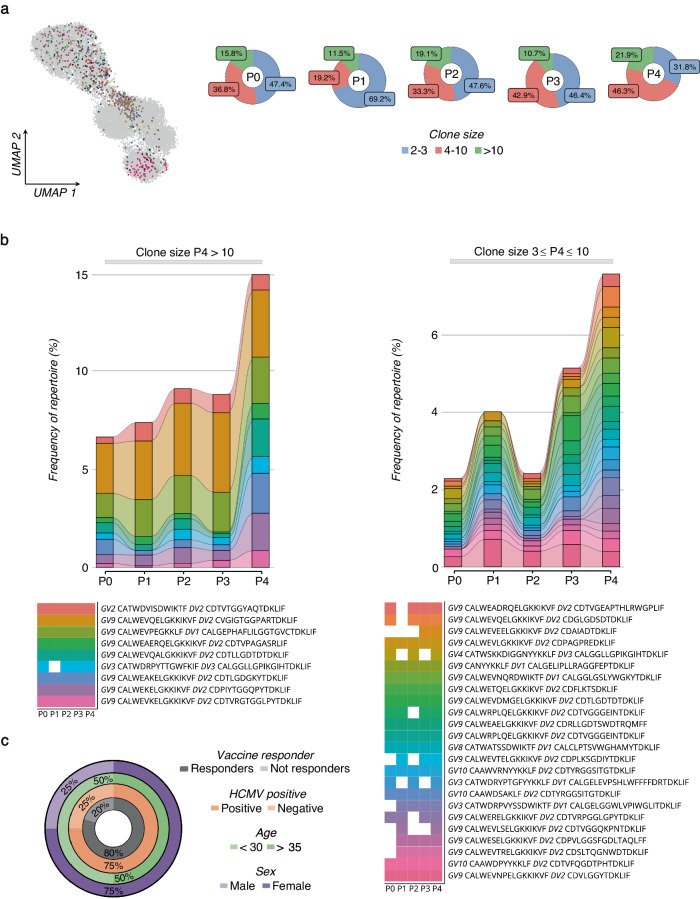
Expansion of γδ TCR clones upon SARS-CoV-2 vaccination. **a** *UMAP* visualization (left panel) of all identified and expanded γδ TCR clones with a cell size ≥2. Each color represents a different clonotype. The pie charts (right panel) show the percentage frequency distribution of the expanded γδ TCR clones grouped by cell sizes across all the time points (P0-P4). **b** The two alluvion plots show the longitudinal tracking of the expanded γδ TCR clones, grouped by the cell size, ≥3 - ≤10 (left panel) and >10 (right panel). Each stratum represents a unique γδ TCR clonotype highlighted by a different color. The time point distribution (P0-P4) and sequence of each clonotype are reported below. **c** The nested pie chart shows the frequencies (%) of subjects who responded to the vaccine with clonal expansion among all the analyzed individuals (inner gray chart). The distribution of HCMV seropositive status, age, and sex among responsive subjects is represented in the colored charts, progressing from the innermost to the outermost circles, which are colored in orange, green, and violet, respectively.

A key question that has emerged was the timescale for the formation and persistence of the γδ-TCR repertoire following vaccination. To address this question, we identified all clonotypes numerically expanded during P1-P4 in relation to their basal level at P0, and which lasted 3 months after the recall of the vaccine (P4). In total, we identified 59 clones that met these criteria (Fig. 7a). The numerical cell-size distribution among expanded clones showed that the first vaccine dose prevalently induced the expansion of relatively small-sized clones (2–3 cells). In contrast, the booster prompted the expansion of larger 4–10 cell-size clones, which further increased over time (Fig. 7a and Supplementary Fig. 6a). The expansion of small cell-size clones was in line with a study on αβ T lymphocyte showing that mRNA-based SARS-CoV-2 vaccination led to the expansion of small cell-size clones (2–3 cells) compared to natural infection^43^. Importantly, this specific clonotype-tracking revealed that the first dose resulted in a limited clonal expansion that declined after 2 weeks. Instead, the second dose induced a higher clonal expansion which peaked 3 months later (Fig. 7b). While vaccine-preexisting clones could also expand, the prevalence (60%) of the expanded clones originated from unique clonotypes. We found the clonal expansion in 80% of analyzed individuals, including both females and males with ages ranging from 26 to 51 years old, and with both HCMV-seropositive and HCMV-seronegative status (Fig. 7c). Among the expanded clones, there was an enrichment (17% vs 1%) of public clonotypes shared by more individuals, with expansion occurring under subject-specific circumstances. Comparing the *DV*/*GV* gene usage in expanded clones, we found dominancy (76%) of *GV9*/*DV2* paired clones that, as expected, show a high phylogenetic relationship compared to other *DV1* and *DV3* clones (Supplementary Fig. 6b). Importantly, the dissimilarity among individually expanded *GV9*/*DV2* clones also resulted in significant genetic distances based on amino acid sequence homology, indicating the expansion of unique *GV9*/*DV2* variants (Supplementary Fig. 6c). Additionally, vaccination was associated with an increase in the length of the complementary-determining region 3 (*CDR3*), particularly for *CDR3*δ (Supplementary Fig. 6d).

Hence, the γδ-TCR repertoire displayed a high clone-specific sensitivity to SARS-CoV-2 vaccination.

### Multiple signals converge on memory Vδ2 T cell development following the SARS-CoV-2 vaccination

**Fig. 8:**
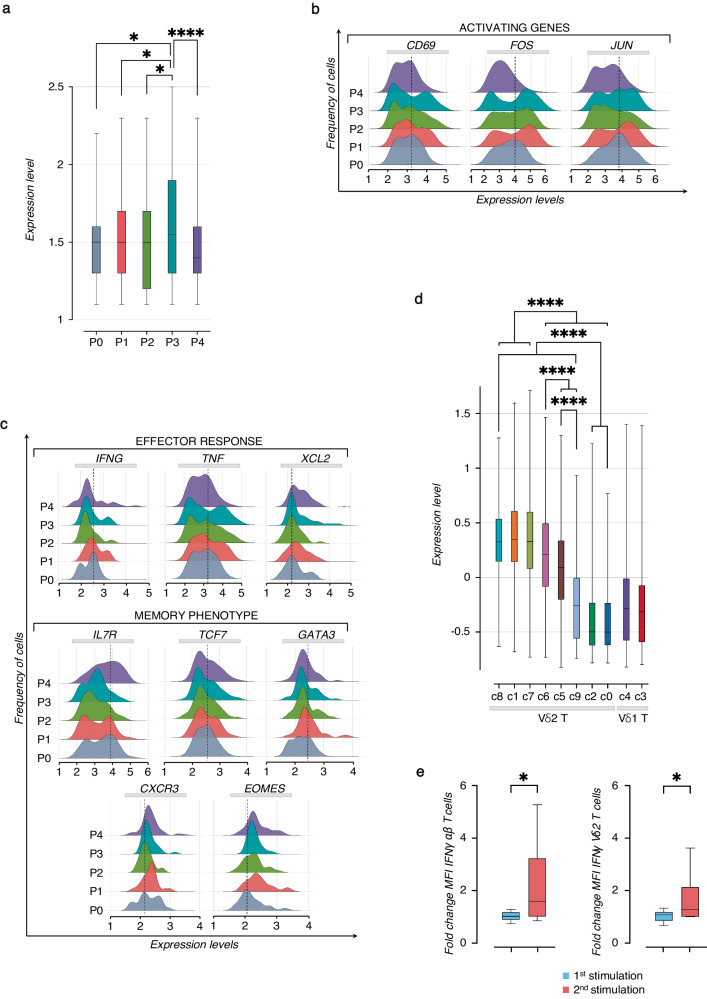
Establishment of memory-like response of Vδ2 T cells following repeated SARS-CoV-2 vaccination and peptide in vitro stimulation. **a** The box plot shows the median values, represented by a line across the boxes, and vertical bars of the min-max range distribution of the cycling gene score calculated in the expanded γδ TCR clones at different time points (P0-P4). For the statistical analysis, the unpaired parametric *ANOVA* test was used, and only significant *p* value*s* (*) are shown: *P < 0.05 and ****P < 0.0001. **b**, **c** The ridge plots show the expression levels (x-axis, UMI-log) and the frequency of cells (y-axis) of selected genes on the expanded γδ TCR clones at different time points (P0-P4), divided for activation, effector and memory signature. The dotted line indicates the gene expression level corresponding to the highest cells’ frequency at the baseline (P0). **d** The box plot shows the median values, represented by a line across the boxes and vertical bars of min-max range distribution, of T_CM_-associated gene score (*IL7R*, *TCF7*, *GATA3*, *CXCR3*, *EOMES*) identified in the expanded γδ TCR clones and calculated for all γδ T cell clusters (c0-c9). For the statistical analysis, the unpaired parametric *ANOVA* test was used, and only significant *P* value*s* (*) are shown: ****P < 0.0001. **e** Functional analysis of Vδ2 T cell response upon SARS-CoV-2 Prot_S peptides stimulation in vitro. The box plots show the fold change in IFNγ expression (MFI) analyzed by flow cytometry, comparing the first and second stimulations with PepTivator® SARS-CoV-2 Prot_S peptides in αβ T cells (left panel) and Vδ2 T cells (right panel) (*n* = *6*). The graphs show median values represented by a line across the boxes and vertical bars of the min-max range distribution. The fold changes were calculated as the ratio of the MFI value of the 1st stimulation over its unstimulated control and the 2nd stimulation over its un-restimulated control. For the statistical analysis, the paired nonparametric Student *t*-test was used. For all graphs, statistic values are represented as *P* values (*): *P ≤ 0.05 and ****P < 0.0001.

As we mapped the expanded clones onto the *UMAP*, we noted their overlap with the T cell clusters responsive to the vaccine. We then integrated signaling networks of activation and proliferation with the γδ-TCR repertoire changes induced by vaccination. Firstly we applied the known signature of cycling γδ T cells to measure proliferative changes in the expanded γδ-TCR clones (Fig. 8a)^37^. Importantly, we found the peak of proliferation occurring after the second vaccine dose. This was also supported by higher levels of G2M phase cell cycle genes at P3 (Supplementary Fig. 7a and Supplementary Table 2). Subsequently, we linked the transcriptional profile of γδ T cell activation to the T_CM_ signature and vaccine stimulation in the identified clones. Following each vaccine dose, we observed an increased expression of genes like *CD69* and AP-1 TFs (*FOS* and *JUN)* (Fig. 8b), confirming the activation signature of the most responsive clusters found in Fig. 2b. This clonal activation correlated with the acquisition of effector mediators (*FNG*, *TNF*, *XCL2*) and the previously identified (Fig. 4) T_CM_ signature (*IL7R*, *TCF7*, *GATA3*, *CXCR3*, *EOMES*) that persisted at P4 (Fig. 8c). Importantly, by applying this memory signature to calculate its enrichment score in different γδ T cell clusters, we confirmed in vaccine-responsive clones the prevalence of T_CM_ immune phenotypes (Fig. 8d).

Overall, our data strongly support the development of T_CM_ Vδ2 T cells as result of SARS-CoV-2 vaccination.

To provide additional evidence for the Vδ2 T cell memory response identified through scRNA-seq analyses, we performed in vitro experiments by measuring the response of circulating Vδ2 T cells isolated from healthy donors upon stimulations with SARS-CoV-2 Spike-peptides (Supplementary Fig. 7b, c). There was significant donor-to-donor variation in the Vδ2 T cell response, however, we observed that cells after the second exposure to Spike-peptides exhibited slightly increased production of IFNγ compared to the first stimulation. In concordance, we detected an improved reactivity of αβ T cells to Spike-peptides following repeated stimulation, resulting in higher IFNγ production (Fig. 8e and Supplementary Fig. 7d).

## Discussion

Understanding the effects of SARS-CoV-2 vaccine exposures on various T cell compartments is crucial for unveiling an effective T cell memory response against SARS-CoV-2. This knowledge can help to determine susceptibility to subsequent infections and enhance vaccination outcomes. To address this, we conducted a comprehensive analysis of γδ T cell response longitudinally following SARS-CoV-2 vaccine exposure. Our findings, at single-cell resolution, reveal that *BNT162b2* vaccination triggers the activation and expansion of Vδ2 T cells, promoting their functional maturation and memory development.

Vδ2 T cells have been previously described for their protective role against the first SARS-CoV infection, characterized by their expansion, IFNγ production, and cytolytic activity against infected cells^21^. Studies, during the early years of the SARS-CoV-2 pandemic, also highlighted the impact of SARS-CoV-2 infection on activation of γδ T cell activation^44–49^. Furthermore, changes in the differentiation statuses of Vδ2 T cells were observed, suggesting their contribution to the immune responses against SARS-CoV-2^50^. Additionally, better recovery from COVID-19 correlated with a higher frequency of the PB Vδ2 T cells^51^. In vitro studies have also shown inhibitory effects of Vδ2 T cells on SARS-CoV-2 infected cells^29,52^.

Recent study reported the activation of γδ T cell subsets in response to COVID-19 vaccination during pregnancy^30^. Our study explores the detailed dynamics of γδ T cell activation to the vaccine in both healthy men and women adults. We observed distinct responses of Vδ1 and Vδ2 T cells, depending largely on their different TCR-differentiation status associations before vaccination. This aligns with numerous evidences showing intrinsic differences between Vδ1 and Vδ2 T cells, including their activating and inhibitory profiles, functional heterogeneity, and γδ-TCR usage^16–18,37,53^. Factors such as age, gender, HCMV infection, and drug treatment could also influence the response of γδ T cells^24–26,32,53^. However, the relatively small number of individuals in our cohort does not provide sufficient statistical power for assessing correlations between Vδ2 T cell response and these variables.

Our study provides a new framework for exploring human memory Vδ2 T cell features^54^. While, memory proprieties of human Vδ2 T cells were described after Bacillus Calmette–Guérin (BCG)-vaccination^54–58^, Listeria monocytogenes (LM)^59^ and HCMV infection^60^, the differentiation trajectories leading to the memory features of γδ T cells remain poorly understood. Our study, for the first time, tracked temporal transcriptomic changes at single-cell resolution, uncovering a T_CM_ Vδ2 T cell signature established upon SARS-CoV-2 vaccination. This signature is marked by the expression of *IL7R*, *TCF7*, *GATA3*, *CXCR3*, and *EOMES* genes. Moreover, we identified AP-1 TFs as potential drivers for imprinting memory Vδ2 T cells. Interestingly, recent studies evidenced the critical role of AP-1 TFs in establishing memory profiles of adaptive NK cells^61,62^.

Future studies will need to examine the specific mechanism(s) involved in the early activation of Vδ2 T cells induced by the mRNA SARS-CoV-2 vaccine. Molecules recognized by γδ T cells can originate from either the vaccine or cellular components. The *BNT162b2* vaccine is based on genetically engineered mRNA encoding the Spike-protein to induce an immune response with reduced binding to innate immune sensors (e.g., TLRs)^63,64^. Most of the expanded clones we observed were Vγ9Vδ2 T cells, implying the products of the mevalonate pathway^65^. This led us to hypothesize that phosphoantigens (PhAgs) could be a promising target for mRNA-based vaccine adjuvant. In this context, the mevalonate pathway and butyrophilin-BTNA3, which activate Vδ2 T cells through PhAgs recognition, have been proposed as druggable targets for vaccine adjuvant in cancer immunotherapies^66,67^. Alternative activation pathways can involve the TLR cascade, which we detected in immunized γδ T cells. On the other hand, the mRNA SARS-CoV-2 vaccine can induce cytokine-dependent priming of Vγ9Vδ2 T cells, as was observed in unconventional mucosal-associated invariant T (MAIT) cells^68^. In fact, Vγ9Vδ2 T cells can be rapidly activated by IFNα, IL-12, and IL-18^69,70^. We observed that the second dose of vaccine resulted in an improved Vδ2 T cell response in terms of effector response and proliferation. This improved response of Vδ2 T cells was also observed in in vitro experiments where the second stimulation of PBMCs with Spike-peptides led to a higher production of IFNγ. Interestingly, it has been demonstrated that Vγ9Vδ2 T cells can identify *Mycobacterium tuberculosis* (Mtb)-derived peptides, inducing their expansion, IFNγ secretion, and cytotoxic response against BCG-infected cells^71–73^. Therefore, the molecular mechanisms of different antigens recognized by the Vγ9Vδ2 TCR still need to be clarified^71,74,75^.

We acknowledge certain limitations in our study, including the relatively small number of analyzed individuals. Furthermore, the longitudinal tracking of in vivo expanded clones can be affected by various biological factors in combination with vaccination. To minimize this, all participants included in the study were SARS-CoV-2 infection-naïve, showed no disease symptoms and did not receive other vaccines (e.g., the influenza vaccine) during the study. Nevertheless, additional research is necessary to verify the development and the protective function of memory Vδ2 T cells after natural infection following vaccination.

Overall, our findings suggest that the immune response to COVID-19 after mRNA SARS-CoV-2 immunization might benefit from the complementary activation and response of unconventional Vδ2 T lymphocytes in instances where adaptive immunity is failing.

## Methods

### Ethics statement

The enrollable individuals were those who planned to be vaccinated according to the national Italian COVID-19 vaccination program in Humanitas Research Hospital from November to April 2021. The recruitment of healthy volunteers has been performed according to the Declaration of Helsinki and all individuals signed written informed consent. The collection of healthy SARS-CoV-2 vaccinated subjects’ and HDs (buffy coats) PB samples for research purposes has been ethically approved by the Institutional Review Board (IRB) of Humanitas Research Hospital (HRH) (approval 738/20).

### Study design

This study was designed to assess γδ T cell immune responses after immunization with two doses of mRNA COVID-19 (*BNT162b2*)–vaccine in individuals without previous SARS-CoV-2 infection. All participants included in the study did not show any disease symptoms (e.g., fever, cough, etc.) and did not receive other vaccines (e.g., the influenza vaccine). We use paired single-cell RNA-seq and TCR-seq technics to uncover clonal differentiation trajectories of γδ T cells upon repeated *BNT162b2* vaccination. In specific, we performed a longitudinal study on six healthy volunteers (three males and three females, age range 25–50 years old) collecting PB samples 1 day before (P0; *n* = *6*), 3 days after the first vaccine dose (P1; *n* = *6*), 17 days after the first dose (P2; *n* = *5*), 3 days after the second dose (P3; *n* = *6*), and 3 months following the boost (P4; *n* = *5*). In addition, we performed in vitro experiments on 6 HDs (buffy coats).

### PBMCs isolation

PB mononuclear cells (PBMCs) were isolated from buffy coats of HDs (*n* = *6*) or from six healthy vaccinated subjects through Lympholyte^®^-H Cell Separation density gradient solution (Cedarlane Laboratories, Burlington, North Carolina, USA) according to the manufacturer’s instruction. In vitro experiments were performed on freshly isolated PBMCs, with a viability greater than 95%, while single-cell ones on PBMCs previously frozen in Fetal Bovine Serum (FBS) supplemented with 10% of the cryoprotective Dimethyl Sulfoxide (DMSO).

### Anti-SARS-CoV-2 IgG Ab titration

Time points for the anti-SARS-CoV-2 Ab measurement were optimized for an efficient adaptive immune response. The anti-SARS-CoV-2 IgG titration was performed at the Humanitas Research Hospital by a ready-to-use ELISA (enzyme-linked immunosorbent assay) Kit (Ref. no. COV19G.CE) for diagnostic use (DIA.PRO, Diagnostic Bioprobes Srl, Italy) and following the manufacturer’s procedures. The IgG Ab levels were measured by the optical density (OD) 450 nm/620–630 nm. The cut-off OD was evaluated by the formula: Cut-off OD = negative control (NC) + 0.250. The specific concentration of IgG was evaluated by the standards provided in the kit.

### Anti-HCMV IgG Ab titration

The anti-HCMV IgG Ab titration was performed by a ready-to-use ELISA Kit (Ref. no. CMVG.CE; DIA.PRO, Diagnostic Bioprobes Srl, Italy), following the manufacturer’s procedures.

### Flow cytometry

For multiparametric flow cytometry analysis, a standard staining protocol was used. Briefly, cells were first stained for live/dead discrimination by using Zombie Aqua fixable viability kit (BioLegend; San Diego, USA). Subsequently, cells were washed with HBSS-/- supplemented with 2% of FBS (FACS buffer) and incubated with the combination of anti-human monoclonal antibodies (mAbs) for 20 min in the dark at room temperature (RT), washed again, permeabilized and fixed with Cytofix/Cytoperm™ solution (BD Biosciences) for 30 min in the dark at 4 °C, washed with Perm Wash™ buffer 1X (BD Biosciences), incubated with the intracellular mix for 30 min in the dark at 4 °C, and then washed with Perm Wash™ buffer 1X.

In the flow cytometry panel, γδ T cells were identified among the CD3^+^CD19^-^ viable lymphocytes expressing either TCR Vδ1 or TCR Vδ2 and tested for the release of IFNγ (CD3: Company BD, Fluorochrome BUV661, Clone UCHT1, Cat. 565065, Concentration 24 µg/ml; CD19: Company Biolegend, Fluorochrome BV510, Clone HIB19, Cat. 302242, Concentration 12 µg/ml; TCR Vδ1: Company Miltenyi, Fluorochrome PE, Clone REA173, Cat. 130120440, Concentration 50 µg/ml; TCR Vδ2: Company BD, Fluorochrome BUV395, Clone B6, Cat. 743754, Concentration 12 µg/ml, IFN-γ: Company Biolegend, Fluorochrome PE-Cy7, Clone B27, Cat. 506518, Concentration 6 µg/ml).

IFNγ release has been investigated in freshly isolated PBMCs obtained from healthy donors naïve for SARS-CoV-2. Cells were stimulated one or two times with peptides PepTivator^®^ SARS-CoV-2 Prot_S (1 μg/ml, Miltenyi Biotec). For the detection of cytokines, the GolgiPlug™ Protein Transport Inhibitor (BD Biosciences) was used to block intracellular protein transport processes. PBMCs were plated in a U-bottom 96 multi-wells plate at a concentration of 2 × 10^6^ cells/200 µl in RPMI complete with 10% of Human Serum in a humidified atmosphere at 37 °C with 5% of CO_2_ with or without 1 µg/ml of peptides PepTivator^®^ SARS-CoV-2 Prot_S (Miltenyi Biotec) for 4 h to evaluate the response upon the first challenge. To evaluate the response to the second challenge, part of the stimulated PBMCs were maintained in culture with the supplement of IL-2 (200 U/ml, Peprotech) and IL-15 (10 ng/ml, Peprotech), and the second challenge was evaluated in a time frame between 8 and 17 days to peak the highest response.

All samples were acquired by the FACS Symphony A5 flow cytometer (BD Biosciences) and BD FACSDiva^TM^ Software, and FCS files were analyzed on FlowJo^TM^ v10.8.1 software (TreeStar Inc, Ashland, Oregon, USA). Flow cytometry data were compensated by using single stained controls with BD Compbeads (BD Biosciences) conjugated to the specific fluorescent mAb. For accurate flow cytometry practice, all mAbs used for the study were previously titrated^76^.

### scRNA and scTCR library preparation

Libraries for scRNA-seq were prepared using the Chromium Single Cell Platform with a Single-Cell 5′ Library and Gel Bead Kit (10X Genomics, PE-1000006). Thawed PBMC samples were evaluated for viability prior to scRNA-seq analysis, and all were ≥95% viable. Cells were resuspended in a volume equivalent to 10,000 target cells for each sample, and were individually loaded onto a Chromium single-cell controller (10X Genomics) to generate single-cell gel beads-in-emulsion (GEMs). Captured cells were then lysed and the released RNA was barcoded through reverse transcription in individual GEMs. Complementary DNAs (cDNA) were generated and split to generate additional libraries γδ scTCR-seq amplicons. To this end, gene-specific primers^25^ were used within the 5′ regions of the TRGC and TRDC segments for the enrichment of TCR transcripts.

Complementary DNAs were amplified, and the quality was assessed using an Agilent 4200 Tapestation.

The scRNA-seq libraries were sequenced using an Illumina Novaseq 6000 sequencer with a paired-end 150-bp (PE150) reading strategy (performed by CapitalBio Technology) and the scTCR-seq libraries were sequenced on Illumina NextSeq 550 platform.

### Processing scRNA-seq data

The scRNA-seq reads were aligned to the GRCh38 (version refdata-gex-GRCh38-2020-A, 10X Genomics) human genome reference, and UMIs were quantified using Cell Ranger 5.0.0 (10X Genomics). Subsequent analyses were performed using the Python package *Scanpy* v1.8.1 under Python v3.8, if not stated otherwise. Raw data matrices of all samples were merged and cells with fewer than 600 expressed genes or 1200 UMIs, or greater than 8% mitochondrial genes were removed. Data were then log-normalized with a scale factor of 10,000. Highly variable genes (HVGs) were identified using the *Seurat* dispersion-based methods. Principal component analysis (PCA) was performed using HVGs via ARPACK implementation of singular value decomposition. To limit the risk of biases, multiple sample-data integration was performed using the harmonypy package v0.0.6^77^. More in detail, the 28 samples (labeled with a progressive sample_id name) were integrated using the sample_id as key covariate in the formula. Neighbors were identified using the top 50 components of Harmony-corrected PCA embeddings and clustering was performed using the *Leiden* algorithm with an initial resolution of 0.1. Clusters were then embedded in two dimensions by using *UMAP*.

### Cluster marker identification and cell-type annotation

Differential expression (DE) analysis between clusters was carried out to find markers for each of the identified clusters by using the *Wilcoxon rank-sum* test (*Scanpy*). Genes with an FDR-adjusted *P* < 0.05 and expressed by at least 10% of cells in the cluster at a minimum Log_2_-Fold Change of |0.25| were considered significant. A total of 12640 γδ T cells were profiled and annotated based on the expression of canonical cell-type markers. Identified clusters were subjected to the next round of dimensionality reduction and unsupervised clustering, as described above.

### Functional enrichment

Reactome enrichment analysis was performed using the Reactome web tool (https://reactome.org/). The Reactome pathways of cell type were enriched using DEGs with FDR-adjusted *P* < 0.05 and Log_2_-Fold Change > |0.25|. Only enriched terms with FDR < 0.05 were selected as significant and visualized by R package *ggplot2* v3.4.0 under R v4.2.1. Dots are colored by FDR values and sized by the number of DEGs enriched in each pathway.

### Pseudotime ordering

Pseudotime trajectory analysis was performed by Monocle v2.8.0^78^. HVGs identified by *Scanpy* were used as the ordering genes. A trajectory was constructed both by merging unstimulated (P0) and stimulated (P1-P4) cells and separately for unstimulated (P0) and stimulated (P1-P4) cells, including only Vδ2 T cells. The T_N_ Vδ2 T cells (c8) were selected as the root nodes of the trajectory graph. *DifferentialGeneTest()* function was used to test for a significant correlation between gene expression and pseudotime. A gene was defined as significantly associated with pseudotime if its estimated *q* value was lower than 0.01.

### Mathematical definition of the effectorness score

To define the effectorness score, we scaled the pseudotime values to the range [0; 1] that correlate to the gene expression profiles of cells along the pseudotime trajectory (0 = Naïve, 1 = Effector memory). To compare pre-vaccine (P0) to post-vaccine (P1-P4), the pseudotime values of cells within the two conditions were scaled to the range [0; 1] and combined into a single data set. The expression of effectorness-dependent genes was visualized on a heatmap for the two trajectories, using the *plot_pseudotime_heatmap()* function. The cell density along the pseudotime was calculated based on the effectorness score computed on total cells belonging to all the time points.

### Modeling the interaction between effectorness and vaccination

The relationship between gene expression, effectorness, and vaccination was modeled as a linear function that accounts for effectorness (a numeric variable that ranges from 0 to 1), vaccination (a categorical variable with levels P0, P1, P2, P3, P4), and their interaction as specified in the following equation^39^:

$${X}_{i,j}={\beta E}_{j}+{\gamma T}_{j}+{\delta E}_{j}\times {T}_{j}+\alpha +\varepsilon$$

In brief, gene expression was tested with the *lm()* function from R package *Stats* v.3.6.2: **X** is the expression of gene **i** in cell **j** (log of normalized UMIs); **β,** **γ**, and **δ** are the regression coefficients for effectorness (E), time (T), and their interaction (E × T), respectively; **α** and **ε** are the intercept and the random error term which was assumed to follow a normal distribution with a mean of zero. This analysis was restricted to HVGs with the removal of mitochondrial, immunoglobulin, and TCR genes. *ANOVA* (two-sided) was used to test which genes were modulated by effectorness and vaccination acting both independently and jointly. Coefficients and *p* values were computed for each gene and *p* values were corrected for the number of tested genes using the Benjiamini-Hochberg method; a coefficient was defined as significant if its corresponding FDR-adjusted *p* value was lower than 0.05.

### scTCR-seq analysis

The scTCR-seq reads were aligned to the GRCh38 (version refdata-cellranger-vdj-GRCh38-alts-ensembl-4.0.0, 10X Genomics) human genome reference and consensus γδ TCR annotations were performed using Cell Ranger VDJ tools 3.1.0 (10X Genomics). Subsequent analyses were performed using the Python package *Scirpy* v0.10.1^79^ under Python v3.8, if not stated otherwise. TCR quality control was computed by using the *ir.tl.chain_qc()* function; only cells with paired γ and δ chains and cells with paired γ and δ chains plus an additional γ and/or δ chain were incorporated into the analysis. Paired γ and δ chains were detected in 40% of 19,682 cells with annotated γ and/or δ chains. γ and δ chain combinations were visualized via chord diagrams using the circlize package v0.4.8. Clonotypes were defined according to the amino acid sequence identity of γ and δ CDR3 regions. Clonal cells were defined as clonotypes that appeared in at least two cells. TCR annotations were merged with transcriptomics data using *scirpy.pp.merge_with_ir()* function by leveraging the presence of the same cell barcode in both assays. To study TCR clonality, TCR rearrangement data were remapped to the UMAP clusters following dimensionality reduction and clustering.

The alpha diversity of clonotypes was calculated by computing the Normalized *Shannon Entropy* (Shannon index): (1) within clusters and (2) at different ranges of T cell effectorness by using a sliding window technique. In brief, both the Shannon index and the mean effectorness value were computed on cells ordered by increasing effectorness using a sliding window of width 1000. Sliding windows were generated by using the *rollapply()* function provided by R package *zoo* v1.8-10.

Clones were defined as public if their paired γ and δ CDR3 sequences were recovered from at least two donors. γ and δ CDR3 sequence lengths were computed by using the *ir.tl.spectratype()* function.

### Cell cycle and proliferation scores

Specific cell cycle phase score, including G1M, G2M, and S score, were computed on the expanded clones by summing the scaled expression values of a list of cell cycle marker genes (Supplementary Table 2) using the R package *Seurat* v4.1.1 under R v4.2.1^80^. Total proliferation score were calculated based on γδ T cell proliferative signature (*HSPA5, TUBA1A, NFKBIA, EZR, CREM, H3F3B, MKI67*)^25^, and computed on expanded clones by using the *AddModuleScore()* function provided by *Seurat* v4.1.1 under R v4.2.1.

### Statistical analysis

Statistical analyses of flow cytometry and, when specified, of scRNA-seq data were performed using GraphPad PRISM software version 9.3.1 (La Jolla, California, USA). Statistical differences between the two groups of flow cytometry data sets were assessed by the paired nonparametric Wilcox *t-test*. For scRNA-seq data, statistical differences were calculated with paired or unpaired *ANOVA* tests as specified in the legend. Statistically significant *p* values were represented with GraphPad style and summarized with the following number of asterisks (*): *P < *0.05*; **P < *0.01*; ***P < *0.001*; ****P < *0.0001*.

### Reporting summary

Further information on research design is available in the Nature Research Reporting Summary linked to this article.

### Supplementary information

Supplementary Material

REPORTING SUMMARY

## Data Availability

All data relevant to the study are included in the article or uploaded as Supplementary information. The scRNA-seq and scTCR-seq data are available from the Gene Expression Omnibus repository (https://www.ncbi.nlm.nih.gov/geo/), accession code GSE260763. Code used for the analysis and figure generation is available upon request to the corresponding author.
