# Supplementary material for: Expansion of memory Vδ2 T cells following SARS-CoV-2 vaccination revealed by temporal single-cell transcriptomics
Source: NPJ Vaccines. 2024 Mar 20;9:63. doi: 10.1038/s41541-024-00853-9 (PMC10954735; doi:10.1038/s41541-024-00853-9)
Supplement: Supplementary file 1 — Supplementary Material [file 41541_2024_853_MOESM1_ESM.pdf]

SUPPLEMENTAL INFORMATION

Expansion of memory Vδ2 T cells following SARS-CoV-2 vaccination revealed by temporal single-cell transcriptomics

Sara Terzoli[1,2], Paolo Marzano[3], Valentina Cazzetta[1,3], Rocco Piazza[4], Inga Sandrock[5], Sarina Ravens[5], Likai Tan[6], Immo Prinz[5,6], Simone Balin[3], Michela Calvi[1,3], Anna Carletti[3], Assunta Cancellara[1,3], Nicolò Coianiz[1], Sara Franzese[1,3], Alessandro Frigo[1,3], Antonio Voza[2,7], Francesca Calcaterra[1,3], Clara Di Vito[1], Silvia Della Bella[1,3], Joanna Mikulak[1#] and Domenico Mavilio[1,3#*]*

*[#]Shared last authorship; *Corresponding authors*

[1]Laboratory of Clinical and Experimental Immunology, IRCCS Humanitas Research Hospital, Rozzano, Milan, Italy;

[2]Department of Biomedical Sciences, Humanitas University, Pieve Emanuele, Milan, Italy;

[3]Department of Medical Biotechnology and Translational Medicine, University of Milan, Milan, Italy;

[4]Department of Medicine and Surgery, University of Milan-Bicocca, Monza, Italy;

[5]Institute of Immunology, Hannover Medical School (MHH), Hannover, Germany;

[6]Institute of Systems Immunology, Hamburg Center for Translational Immunology (HCTI), University Medical Center Hamburg-Eppendorf, Hamburg, Germany;

[7]Department of Biomedical Unit, IRCCS Humanitas Research Hospital, Rozzano, Milan, Italy.

LIST OF CONTENTS

Our study is supported by supplemental information that include:

Page 4-5: Supplemental Figure 1. scRNA-seq analysis in the SARS-CoV-2 vaccinated subjects (related to Figure 1)

Page 6-7: Supplemental Figure 2. Identity of the specific γδ T cell cluster activation following SARS-CoV-2 vaccination (related to Figure 2)

Page 8-9: Supplemental Figure 3. SARS-CoV-2 vaccination shapes the effectorness of Vδ2 T cells (related to Figure 3)

Page 10-11: Supplemental Figure 4. Specific cluster profiling of γδ TCR repertoire (related to Figure 5)

Page 12: Supplemental Figure 5. Analysis of the public γδ TCR clonotypes (related to Figure 6)

Page 13-14: Supplemental Figure 6. Analysis of the expanded γδ TCR clones upon SARS-CoV-2 vaccination (related to Figure 7)

Page 15-16: Supplemental Figure 7. Increased effector response of Vδ2 T cells following repeated SARS-CoV-2 vaccination and peptide stimulation in vitro (related to Figure 8)

Page 17-18: Supplemental Table 1. Cross-reactive genes (related to Figure 4)

Page 19: Supplemental Table 2. Cell cycle phase genes (related to Supplemental Figure 7a)

Supplemental Figure 1

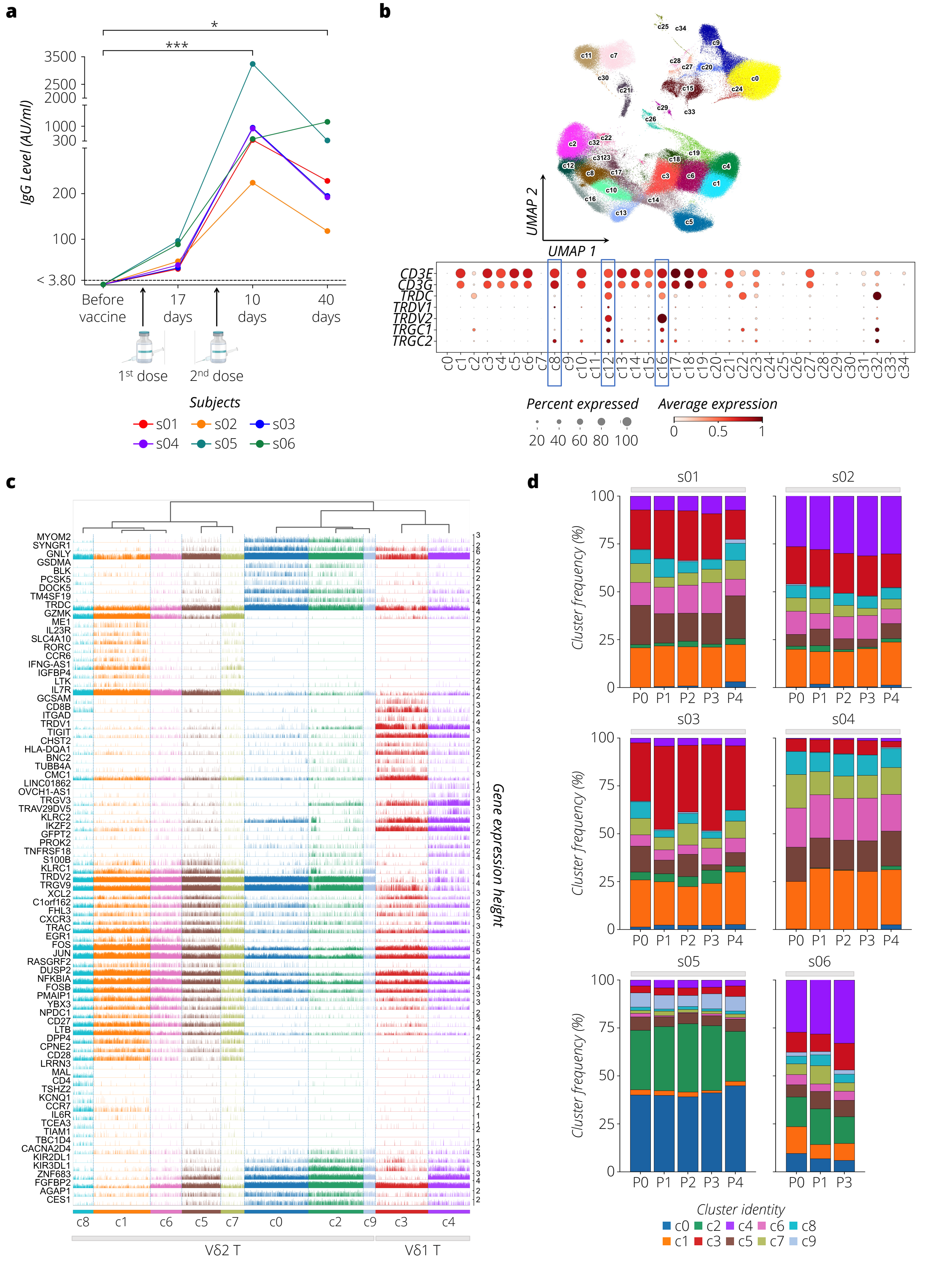

Supplemental Figure 1. Single cell RNA-seq analysis in SARS-CoV-2 vaccinated subjects

(a) Kinetics of anti-SARS-CoV-2 IgG antibody levels in the cohort of vaccinated subjects (s01-s06) at different time points pre- and post-first and second doses of the mRNA-based *BNT162b2* vaccine. For the statistical analysis, the paired *ANOVA* Friedman statistical test was used, and statistic values are represented as *P*-values (*): *$P < 0.05$ and ***$P < 0.001$.

(b) *UMAP* visualization of the integrated single-cell transcriptomes of PBMCs (242,765 cells) identified across all time points (P0-P4), subjects (s01-s06) and cell clusters (c0-c34) after QC filtering. Cell clusters enriched in γδ T cells (c8, c12, and c16), highlighted in blue boxes, were identified based on the expression of known markers (*CD3E, CD3G, TRDC*, *TRDV1*, *TRDV2*, *TRGC1*, and *TRGC2*), and re-clustered for further analysis.

(c) Characterization of specific γδ T cell clusters (c0-c9) obtained after re-clustering. The track plot shows the gene expression height of the top 10 DEGs (rows) for each identified γδ T cell cluster.

(d) The bar plot shows the normalized frequency (%) of γδ T cell cluster (c0-c9) distribution across all analyzed time points (P0-P4) for each subject (s01-s06). The cell cluster’s frequencies were calculated by normalizing against all subject-related cell counts at each time point.

Supplemental Figure 2

a

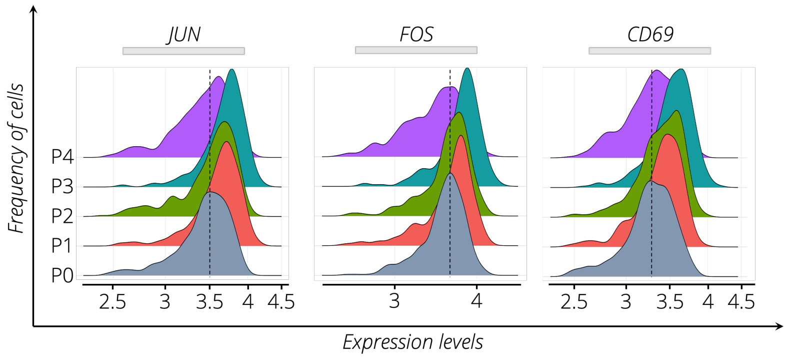

b

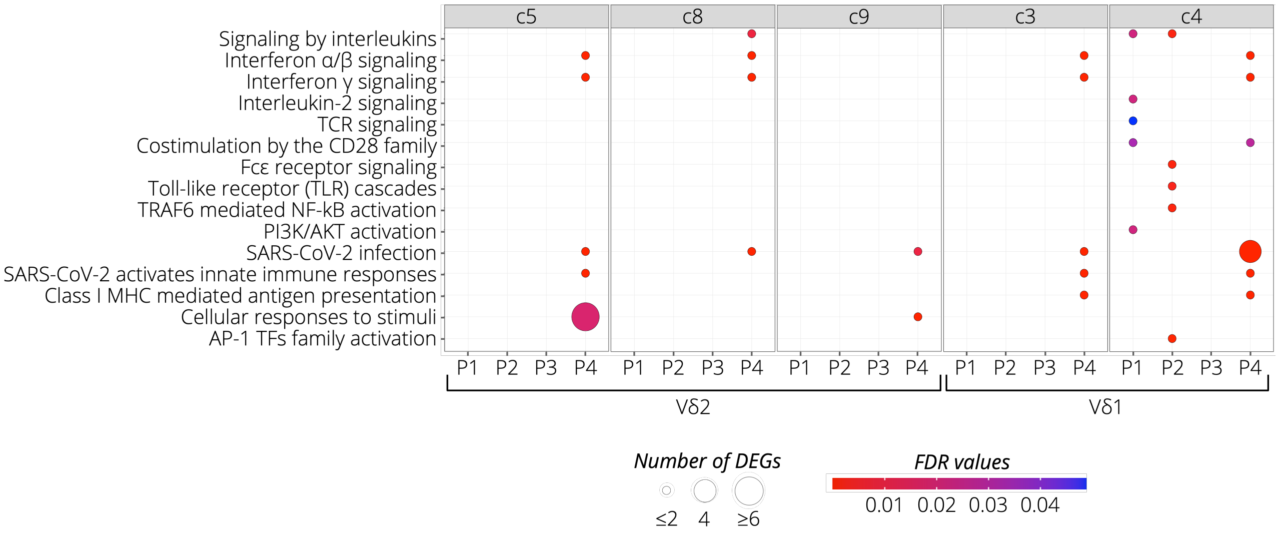

Supplemental Figure 2. Identity of the specific γδ T cell cluster activation following SARS-CoV-2 vaccination

(a) The ridge plots show the expression levels (x-axis, log-UMI) and the frequency of cells (y-axis) for each time point (P0-P4) of three DEGs (*JUN, FOS*, and *CD69)* detected in cluster c1 at P1 *vs* P0. Null gene expression cells were excluded from the analysis. The dotted line highlights the changes in gene expression levels across the different time points (P0-P4) relative to the peak gene expression at the baseline.

(b) The dot plot shows a selection of significantly enriched pathways with FDR values <0.05, identified among DEGs at each time point (P0-P4) for Vδ2 T cell clusters (c5, c8, and c9) and for Vδ1 T cell clusters (c3 and c4) using the *Reactome* pathway browser. Dots are colored based on FDR values and sized according to the number of DEGs enriched in each pathway.

Supplemental Figure 3

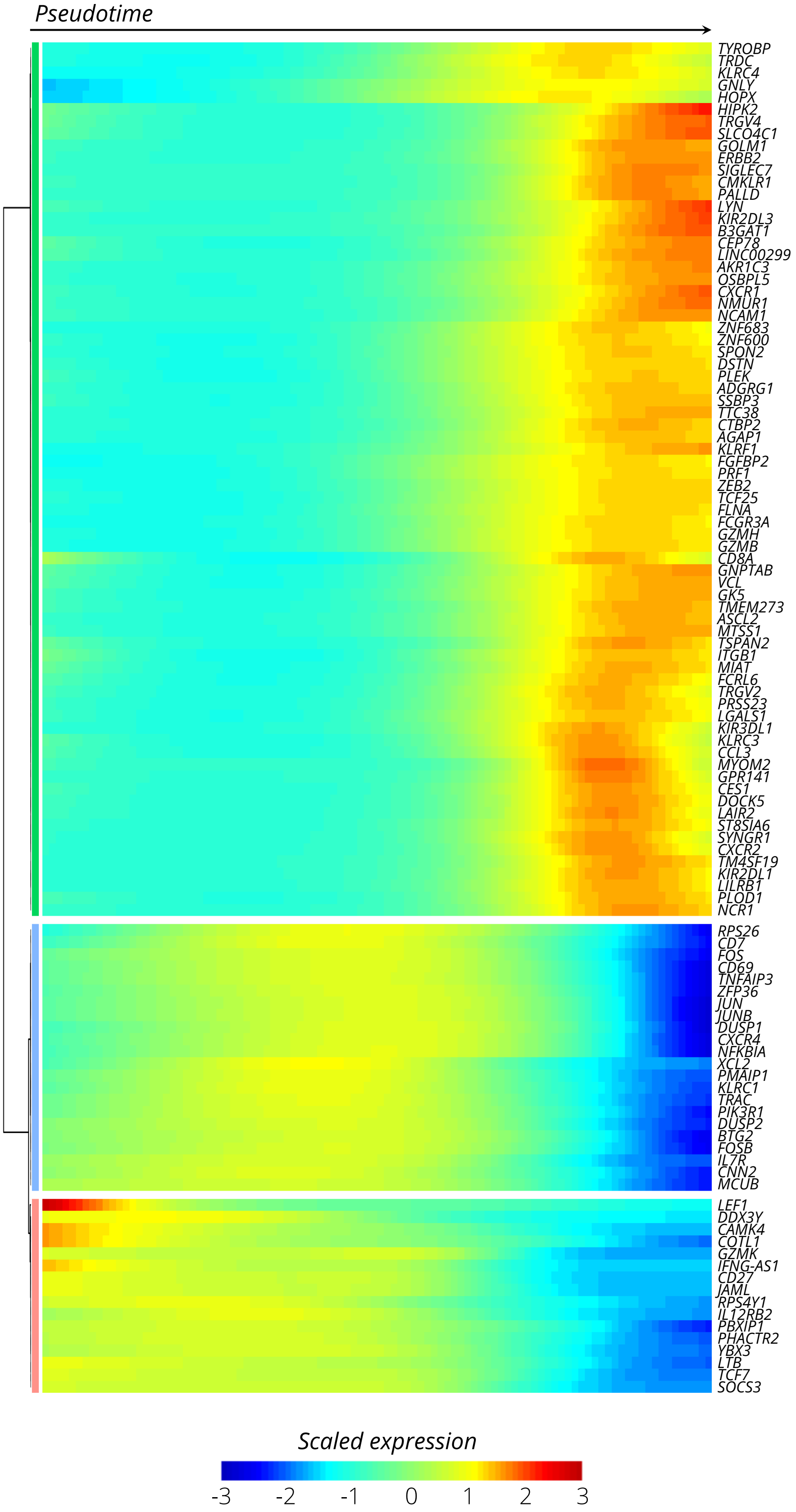

Supplemental Figure 3. SARS-CoV-2 vaccination shapes the effectorness of Vδ2 T cells

Heatmap displaying the top selected 50 genes (*q* value <0.01) variable along the Vδ2 T cell pseudotime trajectory calculated for all time points (P0-P4). The x-axis represents cells ordered by pseudotime (from left to right), and different colors indicate the scaled (Z-scored) expression of each gene in each cell.

Supplemental Figure 4

a

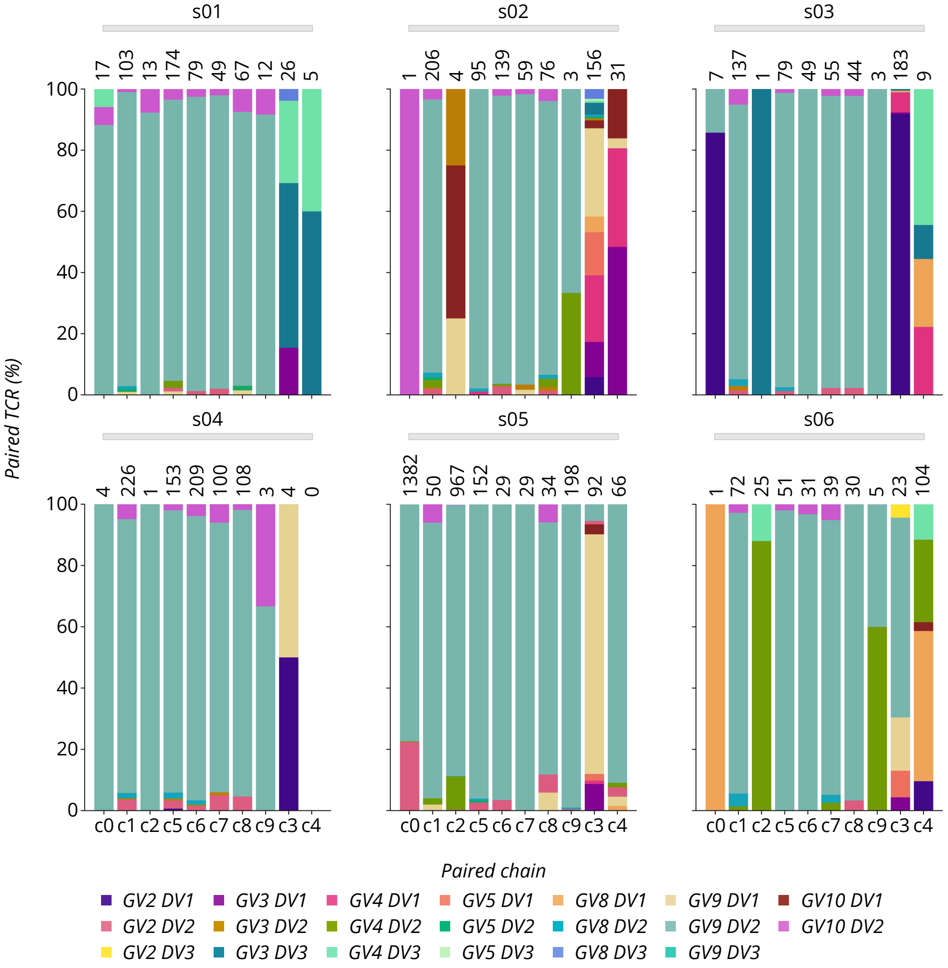

b

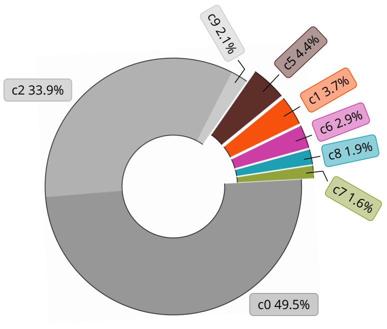

Supplemental Figure 4. Specific cluster profiling of γδ TCR repertoire

(a) The bar plots show the frequency (%) of all paired GV/DV chains in each cluster (c0-c9) per subject (s01-s04). The total numbers of paired γδ TCR chains are indicated at the top of each bar.

(b) The pie chart shows the frequency (%) of GV/DV paired highly expanded clones (≥50 cells) within each cluster of Vδ2 T cells (c0-c2, c5-c9).

Supplemental Figure 5

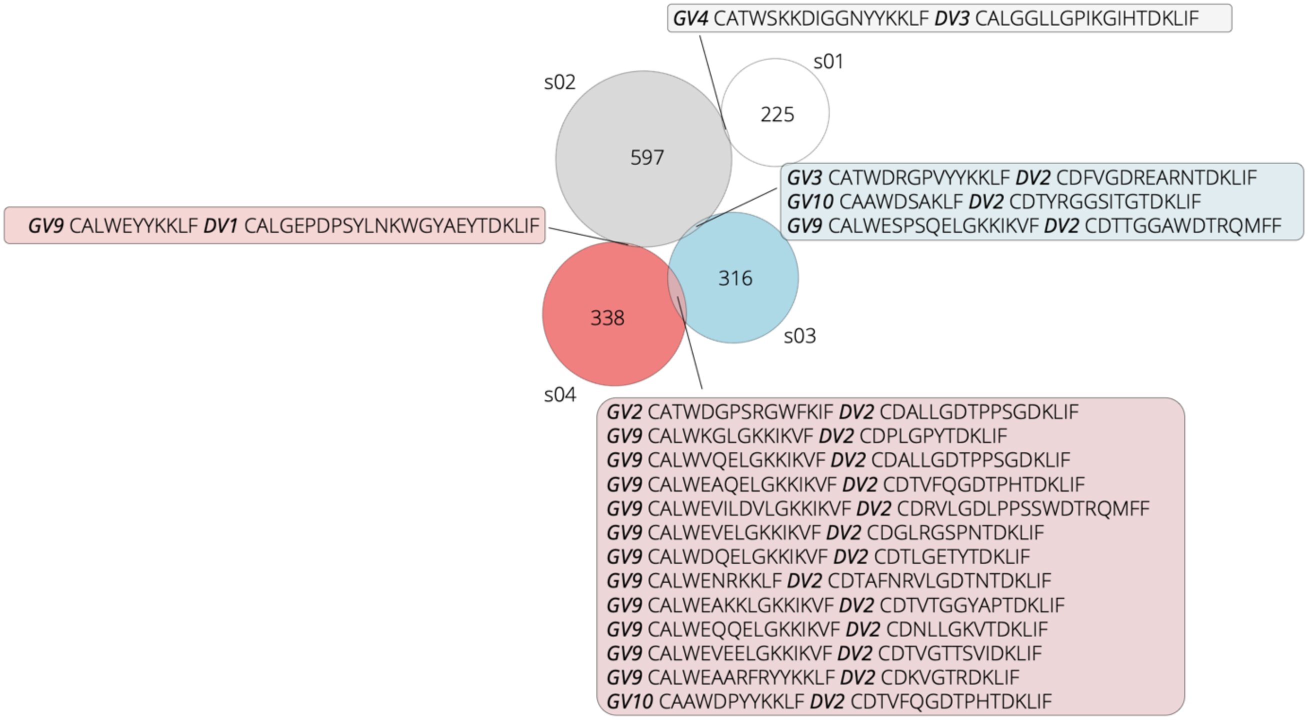

Supplemental Figure 5. Analysis of the public γδ TCR clonotypes

The Venn diagram evidences the number of paired CD3Rγ and CD3Rδ γδ TCR clonotypes that overlap among different subjects (s01-s04).

Supplemental Figure 6

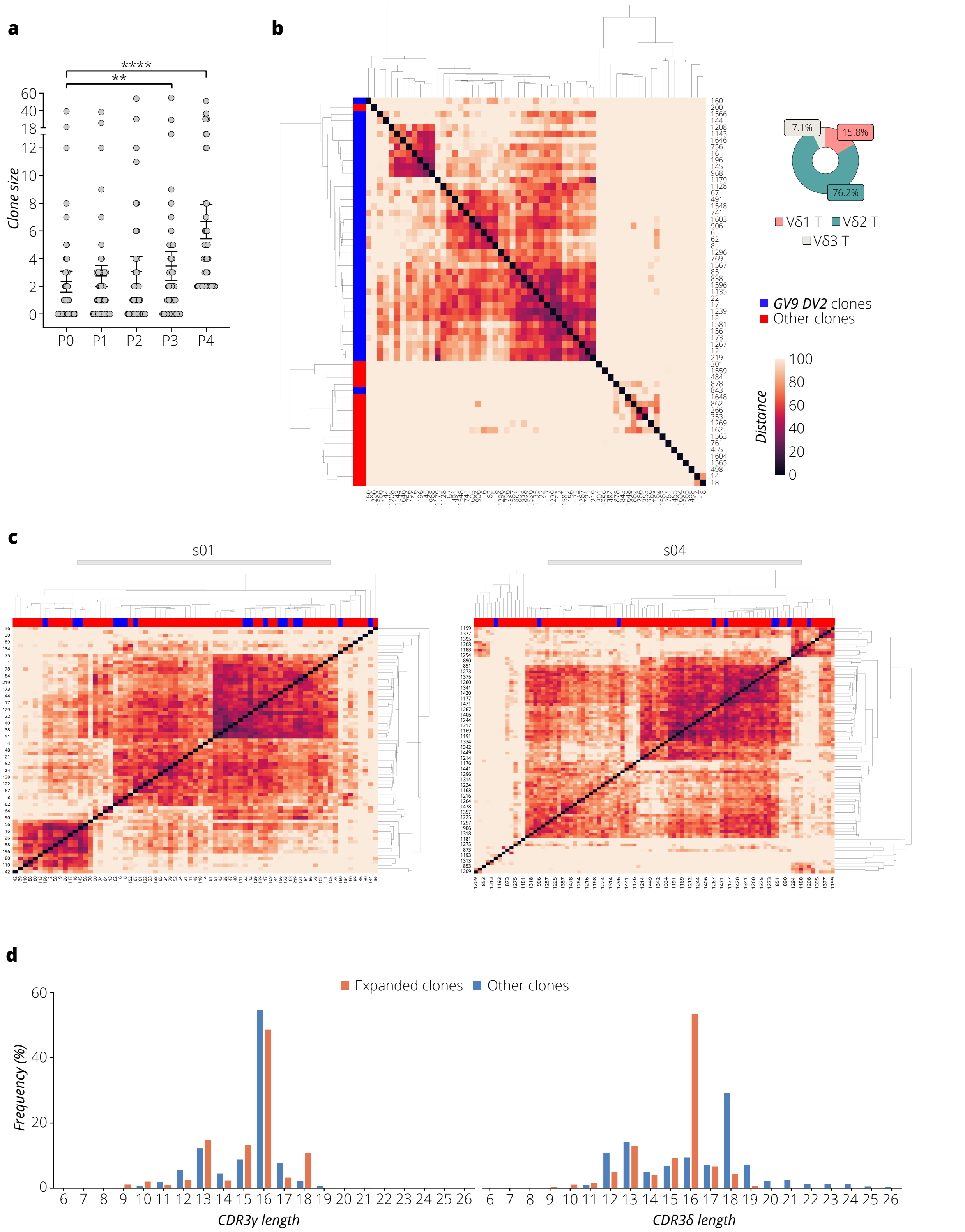

Supplemental Figure 6. Analysis of the expanded γδ TCR clones upon SARS-CoV-2 vaccination

(a) Distribution of γδ-TCR clones with a cell clone size ≥2 across all time points (P0-P4) following vaccination. For the statistical analysis, the unpaired nonparametric *ANOVA* test with Dunn's distribution was used, evaluating all time points (P1-P4) compared to baseline (P0). Statistic values are represented as *P*-values (*): $^{**}P < 0.01$ and $^{****}P < 0.0001$.

(b) The hierarchically-clustered heatmap shows the CD3Rγ and CD3Rδ amino acids sequence distance for expanded γδ TCR clones. Dark red indicates high similarity (low distance), while rose indicates low similarity (high distance). *GV9DV2* clones are highlighted in blue, while other clones are in red. The pie chart shows the frequency of *DV* chains of expanded paired clones.

(c) The example of hierarchically-clustered heatmap shows the CD3Rγ and CD3Rδ amino acids sequence distance for *GV9DV2* clones for subjects s01 and s04. Dark red indicates high similarity (low distance) while rose indicates low similarity (high distance). The *GV9DV2* expanded clones are highlighted in blue, while the total *GV9DV2* clones are in red.

(d) The length percentage frequency distribution (%) of CDR3γ (top) and CDR3δ (bottom) was calculated in the expanded paired γδ TCR clones following vaccination (in red), and in all other identified clones (in blue).

Supplemental Figure 7

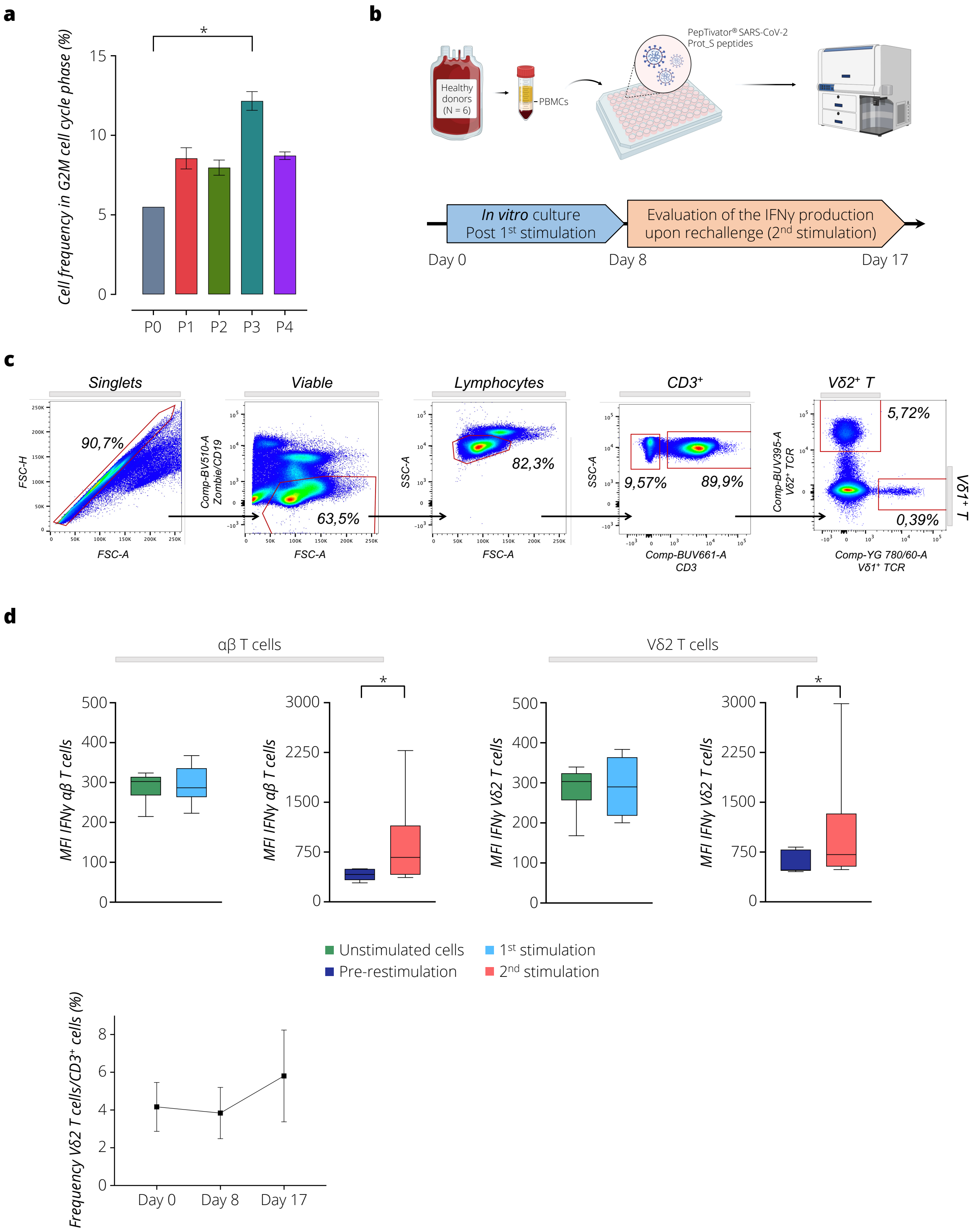

Supplemental Figure 7. Increased effector response of Vδ2 T cells following repeated SARS-CoV-2 vaccination and peptide stimulation *in vitro*

(a) The bar plot shows the percentage (%) of expanded γδ TCR clones in the G2M cell cycle phase. For the statistical analysis, the standard error of the mean (SEM) was calculated according to the absolute cell count, and the unpaired parametric *t-test* was used between each time point (P1-P4) compared to the baseline (P0). Statistic values are represented as *P*-values (*): $*P < 0.05$.

(b) Schematic overview of the *in vitro* experimental design.

(c) Representative flow cytometry dot plots showing the gating strategy used to distinguish the two subsets of γδ T cells, Vδ1 and Vδ2, among all the viable peripheral blood $CD3^+$ lymphocytes.

(d) Functional analyses of Vδ2 and αβ T cell response upon SARS-CoV-2 Prot_S peptides stimulation showing the IFNγ expression (MFI values) after the first and the second stimulation with peptides compared to their respective controls. The frequency of Vδ2 T cells among the $CD3^+$ T cells is reported at each of the three time points analyzed in the experiment. For the statistical analysis, the paired nonparametric *t-test* was used. Statistic values are represented as *P*-values (*): $*P < 0.05$.

Supplemental Table 1

No	Gene	Effectorness adj.p < 0.05	Time adj.p < 0.05	Interaction adj.p < 0.05
1	DUSP1	0E+00	4E-25	8E-93
2	JUN	0E+00	2E-23	1E-83
3	NFKBIA	6E-292	6E-09	2E-80
4	PPP1R15A	6E-149	3E-24	3E-60
5	FOS	0E+00	3E-40	7E-59
6	JUNB	0E+00	2E-39	1E-54
7	GIMAP7	4E-21	6E-33	3E-53
8	CXCR4	0E+00	8E-90	1E-52
9	CD69	0E+00	2E-09	5E-52
10	TNFAIP3	0E+00	4E-22	2E-45
11	ZFP36	0E+00	3E-78	9E-42
12	NR4A2	3E-121	9E-51	1E-40
13	CX3CR1	0E+00	2E-36	7E-38
14	GIMAP5	7E-19	3E-09	2E-34
15	BTG2	2E-199	1E-36	8E-31
16	PIK3R1	0E+00	9E-65	8E-31
17	RBM38	5E-41	6E-62	1E-30
18	TSC22D3	3E-85	4E-07	3E-29
19	FOSB	2E-216	7E-12	5E-29
20	GADD45B	2E-40	2E-35	8E-28
21	AC020916.1	4E-28	4E-03	5E-27
22	CREM	2E-09	1E-48	9E-26
23	KLF6	3E-02	7E-60	1E-24
24	EGR1	5E-66	3E-60	2E-23
25	ZNF331	7E-44	5E-58	9E-20
26	MAP3K8	6E-43	3E-46	4E-19
27	JUND	1E-37	2E-04	4E-19
28	IER2	5E-64	3E-28	2E-18
29	S100A11	9E-11	7E-09	8E-18
30	PLEK	7E-173	2E-07	1E-17
31	PDE4D	1E-38	2E-24	4E-17
32	MAFF	6E-38	1E-30	2E-16
33	DUSP2	0E+00	6E-97	2E-16
34	FOSL2	4E-11	3E-27	3E-16
35	TENT5C	6E-146	5E-37	7E-16
36	YPEL5	9E-30	3E-21	8E-16
37	SERTAD1	3E-23	2E-27	9E-16
38	PMAIP1	1E-155	2E-14	9E-16
39	NFKBIZ	1E-120	1E-17	3E-15
40	DNAJB1	1E-160	6E-30	6E-15
41	PER1	2E-26	1E-13	7E-15
42	IER5	1E-79	3E-20	7E-15
43	PTPN6	4E-02	4E-02	1E-14
44	SPON2	0E+00	6E-08	1E-13
45	PRF1	0E+00	4E-28	2E-12
46	DUSP5	2E-52	8E-16	2E-12
47	AC245014.3	3E-81	2E-09	1E-11
48	ZFP36L1	1E-10	9E-03	1E-11
49	RPS4Y1	0E+00	1E-34	1E-11
50	AC044849.1	6E-51	2E-05	1E-11
51	IFNG	2E-59	2E-08	1E-11
52	ACTB	3E-90	5E-03	2E-11
53	SNX18	9E-29	6E-03	2E-11
54	PPBP	5E-02	7E-31	9E-11
55	METRNL	3E-10	4E-17	9E-11
56	BCL3	6E-12	3E-13	1E-10
57	LINC01871	3E-32	3E-03	1E-10
58	CISH	9E-14	5E-10	1E-10
59	PIM1	2E-03	2E-14	4E-10
60	PLEKHF1	3E-94	6E-03	1E-09
61	YBX3	4E-160	4E-07	2E-09
62	NFKB1	8E-84	2E-21	2E-09
63	SLFN5	1E-03	1E-12	2E-09
64	DYNLL1	4E-35	3E-04	2E-09
65	MYADM	1E-42	9E-25	2E-09
66	CSRNP1	1E-78	8E-12	2E-09
67	MCL1	1E-55	3E-11	4E-09
68	HOPX	2E-178	8E-03	4E-09
69	HOOK2	5E-94	5E-10	4E-09
70	KIAA2013	3E-31	9E-08	5E-09
71	ZC3H12A	2E-13	6E-14	9E-09
72	AC253572.2	6E-92	6E-04	1E-08
73	FCGR3A	0E+00	2E-07	2E-08
74	TUBA1A	6E-11	8E-09	2E-08
75	TUBB4B	3E-06	2E-09	3E-08
76	S100A4	0E+00	2E-11	3E-08
77	IRF4	1E-29	2E-09	3E-08
78	TSPYL2	5E-77	3E-11	7E-08
79	SOCS3	3E-161	3E-14	2E-07
80	EML4	8E-166	6E-12	2E-07
81	SNHG3	8E-08	2E-04	3E-07
82	STARD3NL	7E-34	6E-04	3E-07
83	EPB41L4A-AS1	2E-09	3E-03	3E-07
84	SH2D3C	1E-12	1E-05	3E-07
85	NR4A3	6E-47	2E-14	3E-07
86	IL7R	0E+00	8E-18	4E-07
87	KLRC2	1E-60	2E-07	6E-07
88	EEF1A1	0E+00	3E-04	6E-07
89	WHRN	6E-18	1E-09	9E-07
90	RGS2	2E-21	2E-03	9E-07
91	UBE2S	1E-39	1E-06	1E-06
92	AKR1C3	2E-186	3E-02	2E-06
93	ARL4A	5E-56	9E-05	4E-06
94	IER5L	7E-15	2E-14	4E-06
95	CXCR3	4E-37	1E-06	4E-06
96	LMNA	3E-10	3E-07	5E-06
97	SOCS1	5E-44	3E-08	6E-06
98	TAGAP	3E-03	1E-10	7E-06
99	DDIT4	1E-17	6E-40	1E-05
100	BHLHE40	5E-43	1E-05	1E-05
101	CAMK4	0E+00	1E-04	2E-05
102	SPN	5E-87	3E-07	2E-05
103	PTGER2	7E-31	2E-07	3E-05
104	UCP2	2E-15	4E-06	4E-05
105	HIST1H3D	1E-26	3E-04	7E-05
106	RPSA	0E+00	5E-04	7E-05
107	SYTL3	9E-04	2E-18	8E-05
108	AC087623.2	2E-11	4E-06	8E-05
109	HIST1H1E	2E-04	2E-15	8E-05
110	ST6GAL1	3E-02	1E-04	1E-04
111	GPR65	2E-04	3E-02	1E-04
112	DDIT3	2E-02	6E-04	1E-04
113	ZFP36L2	0E+00	2E-46	2E-04
114	NFATC2	3E-39	2E-03	2E-04
115	RGCC	2E-88	3E-16	2E-04
116	CD5	1E-67	7E-09	3E-04
117	SMAD7	1E-08	1E-02	3E-04
118	AC012615.1	1E-08	3E-05	3E-04
119	ICAM2	4E-12	1E-02	3E-04
120	SKIL	1E-18	3E-02	3E-04
121	TNF	4E-05	5E-07	4E-04
122	CD83	3E-24	4E-09	4E-04
123	IFRD1	1E-42	4E-08	5E-04
124	IFNG-AS1	6E-130	3E-06	5E-04
125	FASLG	2E-16	6E-04	6E-04
126	ATF3	8E-14	2E-02	7E-04
127	HERPUD1	6E-10	2E-14	7E-04
128	DDX3Y	1E-127	1E-07	8E-04
129	GPR183	5E-114	2E-05	9E-04
130	PHLDA1	5E-10	1E-02	1E-03
131	HSPA1A	3E-13	9E-05	1E-03
132	S100A9	4E-03	7E-07	1E-03
133	LPAR6	3E-03	8E-06	2E-03
134	KIR2DL3	2E-106	6E-04	2E-03
135	PRDM1	1E-32	2E-09	2E-03
136	TNFRSF1A	5E-13	4E-04	2E-03
137	TRAC	0E+00	3E-13	2E-03
138	ODC1	9E-10	1E-02	2E-03
139	CCDC59	1E-08	4E-05	2E-03
140	PDE3B	3E-80	6E-06	2E-03
141	H2AFX	3E-11	4E-07	2E-03
142	SLC1A5	2E-14	4E-02	3E-03
143	TCF7	0E+00	6E-04	3E-03
144	SLAMF6	1E-08	5E-02	3E-03
145	LIMK1	5E-04	4E-03	4E-03
146	RPL8	0E+00	8E-04	5E-03
147	CEMIP2	2E-02	1E-07	6E-03
148	PUDP	4E-02	3E-02	6E-03
149	ITPRIP	4E-12	2E-04	6E-03
150	RPLP1	0E+00	2E-14	6E-03
151	CD320	1E-88	2E-03	9E-03
152	GABARAPL1	6E-12	9E-05	1E-02
153	PRSS23	0E+00	5E-05	1E-02
154	HBB	2E-04	9E-10	1E-02
155	CCDC88C	5E-54	6E-10	1E-02
156	AP002387.2	3E-04	5E-03	1E-02
157	ARID5A	5E-06	2E-04	1E-02
158	POU2F1	8E-03	5E-04	1E-02
159	GADD45G	1E-07	7E-05	1E-02
160	TCF25	2E-163	7E-03	2E-02
161	KLRC4	3E-207	6E-03	2E-02
162	CD55	7E-140	2E-04	2E-02
163	AC025164.1	2E-23	1E-02	2E-02
164	AC004865.2	2E-35	5E-05	2E-02
165	PTGER4	1E-26	9E-09	2E-02
166	HIST1H4C	2E-20	2E-03	2E-02
167	AREG	9E-30	3E-06	2E-02
168	ID1	8E-13	3E-12	2E-02
169	TENT4B	2E-06	5E-06	3E-02
170	AC004687.1	7E-15	3E-03	3E-02
171	TNFSF14	4E-04	1E-03	3E-02
172	IRS2	2E-38	4E-06	3E-02
173	EOMES	1E-09	7E-08	3E-02
174	GATA3	8E-25	5E-04	3E-02
175	ITGAM	3E-107	7E-06	3E-02
176	PLEKHG3	9E-44	3E-02	4E-02
177	XCL2	4E-120	8E-04	4E-02
178	HDDC2	5E-22	4E-02	4E-02
179	PTGDR	2E-09	1E-04	5E-02

Supplemental Table 1. Cross-reactive genes

This table provides a list of genes whose expression was significantly modulated by effectorness and vaccination acting independently, as well as jointly due to their cross-reactive effect. Gene expression was modeled as a function of effectorness, time, and their interaction using a linear regression with interaction terms. We used *ANOVA* (two-sided) to test which gene are modulated by effectorness and vaccination acting both independently and jointly. The analysis was restricted to highly variable genes, with the removal of mitochondrial, immunoglobulin, and *TCR* genes. We report the FDR adjusted *P (adj.p)*-value for any gene with a value <0.05. Columns correspond to gene name and *adj.p-value* for effectorness, time and effectorness-time interaction effects.

Supplemental Table 2

**Cell cycle phase genes**
MCM5, PCNA, TYMS, FEN1, MCM2, MCM4, RRM1, UNG, GINS2, MCM6, CDCA7, DTL, PRIM1, UHRF1, MLF1IP, HELLS, RFC2, RPA2, NASP, RAD51AP1, GMNN, WDR76, SLBP, CCNE2, UBR7, POLD3, MSH2, ATAD2, RAD51, RRM2, CDC45, CDC6, EXO1, TIPIN, DSCC1, BLM, CASP8AP2, USP1, CLSPN, POLA1, CHAF1B, BRIP1, E2F8, HMGB2, CDK1, NUSAP1, UBE2C, BIRC5, TPX2, TOP2A, NDC80, CKS2, NUF2, CKS1B, MKI67, TMPO, CENPF, TACC3, FAM64A, SMC4, CCNB2, CKAP2L, CKAP2, AURKB, BUB1, KIF11, ANP32E, TUBB4B, GTSE1, KIF20B, HJURP, CDCA3, HN1, CDC20, TTK, CDC25C, KIF2C, RANGAP1, NCAPD2, DLGAP5, CDCA2, CDCA8, ECT2, KIF23, HMMR, AURKA, PSRC1, ANLN, LBR, CKAP5, CENPE, CTCF, NEK2, G2E3, GAS2L3, CBX5, CENPA.

Supplemental Table 2. Cell cycle phase genes

This table provides the list of marker genes specific to G1M, G2M, and S phases used to calculate cell cycle phase score to expanded γδ TCR clones (related to Supplemental Figure 7a)
